# The long noncoding RNA lnc-EGFR stimulates T-regulatory cells differentiation thus promoting hepatocellular carcinoma immune evasion

**DOI:** 10.1038/ncomms15129

**Published:** 2017-05-25

**Authors:** Runqiu Jiang, Junwei Tang, Yun Chen, Lei Deng, Jie Ji, Yu Xie, Ke Wang, Wei Jia, Wen-Ming Chu, Beicheng Sun

**Affiliations:** 1 Liver Transplantation Center of the First Affiliated Hospital and Collaborative Innovation Center For Cancer Personalized Medicine, Nanjing Medical University, Nanjing, Jiangsu Province 210029, P.R. China; 2 Department of Microbiology and Immunology, Nanjing Medical University, Nanjing, Jiangsu Province 210029, P.R. China; 3 Metabolomics Shared Resource, University of Hawaii Cancer Center, 701 Ilalo Street, Honolulu, Hawaii 96813, USA; 4 Cancer Biology Program, University of Hawaii Cancer Center, 701 Ilalo Street, Honolulu, Hawaii 96813, USA

## Abstract

Long noncoding RNAs play a pivotal role in T-helper cell development but little is known about their roles in Treg differentiation and functions during the progression of hepatocellular carcinoma (HCC). Here, we show that lnc-epidermal growth factor receptor (EGFR) upregulation in Tregs correlates positively with the tumour size and expression of EGFR/Foxp3, but negatively with IFN-γ expression in patients and xenografted mouse models. Lnc-EGFR stimulates Treg differentiation, suppresses CTL activity and promotes HCC growth in an EGFR-dependent manner. Mechanistically, lnc-EGFR specifically binds to EGFR and blocks its interaction with and ubiquitination by c-CBL, stabilizing it and augmenting activation of itself and its downstream AP-1/NF-AT1 axis, which in turn elicits EGFR expression. Lnc-EGFR links an immunosuppressive state to cancer by promoting Treg cell differentiation, thus offering a potential therapeutic target for HCC.

## Abstract (web summary)

The role of long noncoding RNAs in regulating T-cell differentiation within the tumour microenvironment is unclear. Here the authors identify a lncRNA that, through direct interactions with EGFR, promotes T-regulatory cell differentiation within the microenvironment of hepatocellular carcinoma, thus promoting tumour growth via immune suppression.

Hepatocellular carcinoma (HCC) is one of the major malignant tumours worldwide^1^,^2^. Because it is often diagnosed at an advanced stage, a large proportion of HCC patients displays intrahepatic metastasis or postsurgical recurrence, with a poor 5-year survival rate^3^. The development of HCC is believed to be associated with Hepatitis B virus and Hepatitis C virus infections in most patients in the Chinese population^4^. The virus-initiated tumorigenic process often follows from or accompanies long-term symptoms of chronic hepatitis, inflammation, and cirrhosis^5^,^6^. The Hepatitis B virus-infection-triggered inflammatory and/or fibrotic processes, involving extensive cytokine/chemokine production/activation and leukocyte infiltration, are believed to create a microenvironment that favors the development of HCC^7^.

Tumour-infiltrating lymphocytes (TILs) and peripheral blood lymphocytes (PBLs) are two major components of the HCC-associated immune microenvironment^8^,^9^. TILs are considered manifestations of the host immune reactions against cancer^10^,^11^. Patients with a prominent lymphocyte infiltration, especially T lymphocytes, who undergo resection for HCC, have reduced recurrence and better survival^9^. On the other hand, the TILs and PBLs from patients with advanced-stage cancer exert a poor immune response^12^. This tumour-induced immunosuppression includes diminished responses to recall antigens, reduced proliferative T-cell responses, the loss of cytokine production, and defective signal transduction in T cells and natural killer (NK) cells^8^. Moreover, increased apoptotic CD8^+^ T cells were found in PBLs isolated from cancer patients and mice bared with tumours^13^.

Recent studies have demonstrated increased populations of regulatory T cells (Tregs) in the TILs of patients with ovarian cancer^14^, lung cancer^15^, breast cancer^16^ and oesophageal cancer^17^. Tregs are associated with the invasiveness of HCC and the intratumoral balance of regulatory and cytotoxic T cells, and are a promising independent predictor of recurrence and survival in HCC patients^9^. Within the tumour microenvironment, Foxp3-expressing Tregs, which normally function as a dominant inhibitory component in the immune system to actively maintain self-tolerance and immune homoeostasis through suppression of various immune responses, have been found to be co-opted by tumour cells to escape immune surveillance^18^,^19^.

Whole-transcriptome analyses have revealed that a new class of non-protein-coding transcripts designated ‘long noncoding RNAs’ (lncRNAs), are transcribed from a large proportion of the human genome^20^,^21^. LncRNAs have been shown to play a crucial role in the development of human carcinomas and congenital diseases^22^,^23^. Notably, the involvement of lncRNAs in the human immune system, which includes T cells, dendritic cells (DCs) and macrophages, has recently been reported^24^,^25^. For example, lncRNA *Tmevpg1* is specifically expressed by the Th1 subset of cells, via a T-BET-dependent mechanism, and is necessary for the efficient transcription of *ifng* by the Th1 subset^26^, and downregulation of linc-MAF-4 skews T-cell differentiation toward the Th2 phenotype^27^.

In this study, we elucidate the impact of lncRNAs in linking Tregs and HCC. High-throughput screening was used to investigate the transcriptomic associations between lncRNAs and mRNAs in the TILs of HCC patients. A specific Lnc-epidermal growth factor receptor (EGFR) was identified and found highly expressed in Tregs. Its function in Tregs as a tumour promoter and the related mechanisms are examined. The results indicate that lnc-EGFR is a potential enhancer of EGFR and its downstream AP-1/NF-AT1 axis within T cells thus to promote immunosuppression in human HCC.

## Results

### Transcriptome comparison between HCC TILs and PBLs

**Figure 1:**
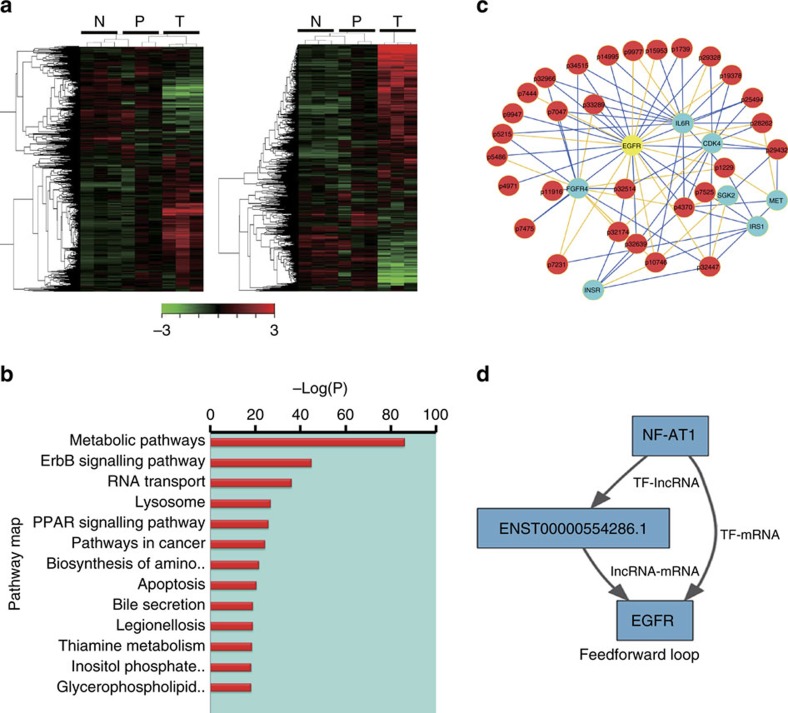
Landscape of mRNA and lncRNA expression in CD4^+^ T cells from TBLs and PBLs of human HCC patients. (**a**) Hierarchical clustering analysis of 1,251 lncRNAs (left panel) and 2,012 mRNAs (right) that are differentially expressed (the threshold of significance was defined by *P*<0.05 (with Student *t*-test) and the false discovery rate) in tumour-infiltrating CD4^+^ T cells (T), paired peripheral blood CD4^+^ T cells (P), and peripheral blood CD4^+^ T cells from healthy controls (N). The clustering tree for lncRNAs and mRNAs is shown at the top. The expression values are shown in shades of red and green, indicating expression above and below the median expression value across all the samples (log scale 10, from –3 to +3), respectively. (**b**) Pathway analysis showing the significant pathways of the differentially expressed protein-coding genes (*P*<0.05 with Student *t*-test). (**c**) A portion of the co-expression network of the candidate lncRNAs and mRNAs representing the significant pathways. A green node represents one mRNA and a red node represents a lncRNA. (**d**) Predicted NF-AT1–lnc-EGFR–EGFR feed-forward loop.

In this study, T cells were extracted from both the tissues and blood of three patients with HCC and the blood of three healthy volunteers. As schematically illustrated in Supplementary Fig. 1, anti-CD3 Magnetic Dynabeads were used to purify the CD3^+^ T cells and the total transcriptome RNA of the samples was used to detect the distribution of both lncRNAs and mRNAs. A differential expression profile of the tumour-infiltrating CD3^+^ T cells was obtained by comparing the microarray signals from the tumour tissue samples with those from the peripheral blood CD3^+^ T cells from both the HCC patients and the healthy volunteers, which showed that 1,251 lncRNAs and 2,012 mRNAs were differentially expressed in TILs with fold changes of 4/0.25. In an unsupervised clustering analysis of all the transcripts, we detected significant differences in the expression signatures of the three sets of samples (Fig. 1a). A principal component analysis (PCA) revealed that the samples derived from the three groups displayed a tendency to form separate clusters when analysed for both lncRNAs and mRNAs (Supplementary Fig. 2a). Interestingly, the peripheral blood CD3^+^ T cells from the HCC patients preferentially associated with the peripheral blood CD3^+^ T cells of the volunteers rather than with the tumour-infiltrating CD3^+^ T cells when the lncRNA and mRNA profiles were merged (Supplementary Fig. 2b).

### Bioinformatic analysis of multilayered feed-forward loops

Using the differentially expressed mRNAs as the input, we analysed the significant pathways associated with them using the Kyoto Encyclopedia of Genes and Genomes (KEGG), BioCarta and Reactome Pathway Database platforms. We identified many enriched pathways, among which the metabolic pathways were most significant (Fig. 1b) but were further investigated elsewhere. In the present study, we mainly focused on the following annotated pathways, including the ERBB and PPAR signalling pathways and cancer pathways. Human anti-CD4 and anti-CD8 antibodies were used to investigate the potential cell populations carrying the aberrantly expressed mRNAs and lncRNAs. We first chose the protein-coding RNAs involved in the above pathways to screen for the differentially expressed mRNAs in the samples used in chip hybridization described above with PCR with reverse transcription (RT–PCR). Twenty mRNAs were identified and their expression levels differed within the three types of samples (TILs and two sets of PBLs); among them was the EGFR, which was most significantly expressed in CD4^+^ T cells but not in CD8^+^ T cells. We next built a lncRNA–mRNA network to identify the lncRNAs that potentially interact with the differentially expressed mRNAs. A further cross-check analysis was conducted between the coexpressed and dysregulated lncRNAs, filtered by the detection density on the microarray in each type of sample. Cells obtained from the same six patients or volunteers were sorted with anti-CD4/anti-CD8 beads, and 49 candidate lncRNAs were chosen for further validation with RT–PCR. We finally identified 28 lncRNAs potentially associated with the selected signalling pathways listed in Supplementary Fig. 2c, d, among which ENST00000554286.1 showed the greatest difference. Both the mRNA and lncRNA screening indicated that the different expression of the candidate RNAs mainly occurred in CD4^+^ T cells, rather than CD8^+^ T cells. On the basis of the normalized signal intensity of the specific genes and lncRNAs with 0.99 as the cutoff value for correlation and a *P* value of 0.01, the closely correlated 20 mRNAs and 28 lncRNAs were used to build a potential target lncRNA–mRNA network. A circled distribution of the correlated lncRNAs and mRNAs revealed that eight mRNAs were strongly associated with the 28 lncRNAs (Fig. 1c). Since transcription factors are often reported to regulate the expression of lncRNAs or mRNAs in human disease, we speculated that multilayered feed-forward loops might exist in the regulatory networks among lncRNAs, mRNAs, and transcription factors. We used the transcription factor analysis software, TFscan, to identify the transcription factors involved. Based on a Match analysis and the correlations between lncRNAs and mRNAs, we identified eight feed-forward loops covering all the candidate mRNAs with the highest degree (Supplementary Fig. 2e). The group with the highest degree and correlation indicated that the transcription of ENST00000554286.1 might be promoted by NF-AT1, which caused the dual activation of EGFR (by both ENST00000554286.1 and NF-AT1). On the basis of this finding, we designated ENST00000554286.1 as ‘lnc-EGFR’ in the subsequent analysis.

### Lnc-EGFR correlates with Treg and cytotoxic lymphocytes

**Figure 2:**
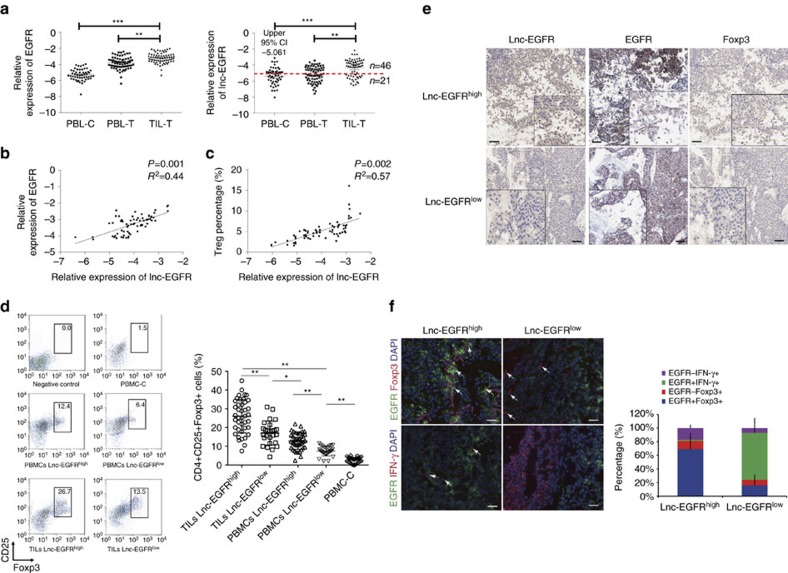
Upregulated lnc-EGFR correlates with the distributions of regulatory T cells and cytotoxic lymphocytes. (**a**) Relatively increased levels of lnc-EGFR and EGFR were confirmed in tumour-infiltrating T cells by comparing them with those in the peripheral blood T cells of HCC patients and healthy controls (Student’s *t*-test). (**b**) Pearson’s correlation analysis was performed to assess the correlation between lnc-EGFR and EGFR (*n*=67). (**c**) The Pearson’s correlation of lnc-EGFR expression and Treg percentage was analysed (*n*=67). (**d**) The percentage of Treg cells in the infiltrated CD4^+^ T cells was determined with flow cytometry in 67 clinical samples, which were further grouped according to the expression of lnc-EGFR. PBLs from the HCC patients and PBLs from healthy subjects were used as the controls, and Foxp3 and CD25 were used for gating CD4. (**e**) The subcellular location and intensity of lnc-EGFR were examined with *in situ* hybridization in sections from HCC patients. The expression of EGFR and Foxp3 was detected with immunohistochemistry in continuous sections from both the lnc-EGFR^high^ and lnc-EGFR^low^ groups (× 100)(*n*=67). (**f**) The expression of EGFR and marker proteins of Tregs (Foxp3) or CTLs (IFN-γ) were detected in human clinical samples. EGFR stained green, Foxp3 or IFN-γ red. Blue 4′,6-diamidino-2-phenylindole (DAPI) staining indicates nuclei (× 100). White arrow indicates positive staining. The quantitative analysis was performed in the left panel (Student’s *t*-test). Each experiment was performed triplicated. Data are presented as means±s.e.m. and analysed with Student *t*-test (*P<0.05, **P<0.01).

To further understand the functions of the candidate lncRNAs and their associated partners, we first conducted an RT–PCR assay to confirm its abnormal expression in the CD4^+^ T cells from 67 individuals with HCC and 52 healthy controls. We found that both lnc-EGFR and EGFR were highly upregulated in tumour-infiltrating T cells compared to the peripheral blood T cells from HCC patients and healthy controls (Fig. 2a and Supplementary Fig. 3). We next investigated the correlation between the significant mRNAs described above and the predicted lncRNA using Pearson’s correlation analysis, and identified a strong correlation between lnc-EGFR and EGFR mRNA (*P*=0.001, *R*^2^=0.44) (Fig. 2b), whereas the other four units displayed weaker correlations (Supplementary Fig. 4). These results suggest that inter-individual differences may lead to significantly different results due to limited sample sizes.

We divided the HCC patients into two groups, lnc-EGFR^high^ and lnc-EGFR^low^, according to the level of lnc-EGFR expressed, using the upper 95% confidence interval (CI) for the peripheral blood CD4^+^ T cells of the volunteers as the cutoff. Analysis of their clinical characteristic revealed that the ectopic expression of lnc-EGFR correlated with tumour size in the HCC patients (Table 1). The distributions of the CD4^+^ T cell subgroups, including Th1, Th2, Th17 and Tregs, were examined in the lnc-EGFR^high^ and lnc-EGFR^low^ patients. The percentage of Tregs, but not Th1, Th2 or Th17 cells, increased as the expression of lnc-EGFR increased (Supplementary Fig. 5). A positive correlation was also identified between lnc-EGFR expression and the percentage of Tregs in HCC patients, suggesting that the presence of lnc-EGFR is associated with the differentiation of Tregs (Fig. 2c). Further flow cytometry analysis, as shown in Fig. 2d and Supplementary Fig. 6a–c, confirmed that lnc-EGFR expression in the CD4^+^ T cells was accompanied by an increased ratio of Treg cells within the tumour microenvironment of either HCC or other human malignant tumours (oesophagus cancer, gastric cancer, lung cancer and colon cancer); CD4^+^-T-cell-specific EGFR also correlated positively with Foxp3, but correlated negatively with interferon γ (IFN-γ), depending on the expression of lnc-EGFR (Fig. 2e,f and Supplementary Fig. 6d,e).

### Lnc-EGFR prevents EGFR ubiquitination by c-CBL

**Figure 3:**
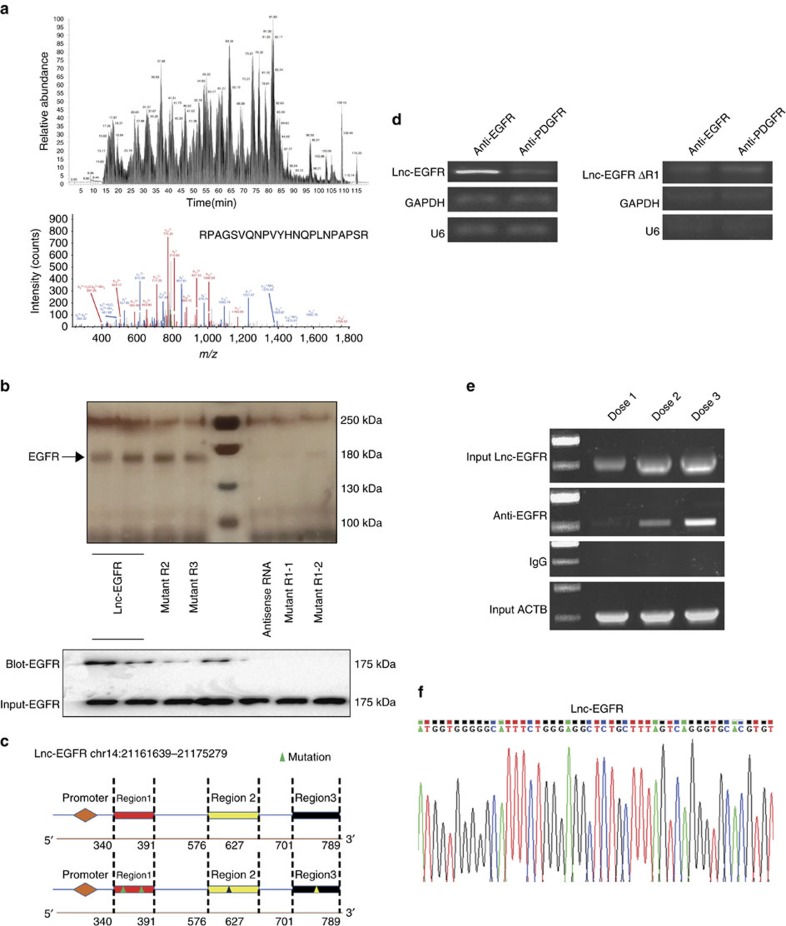
Cytoplasmic lnc-EGFR binds specifically to EGFR. (**a**) Lnc-EGFR RNA pull-down assay was performed. The associated proteins were processed and subjected to Mass Spec. followed by analysis via the Proteome Discoverer program (a1) and the NCBI protein database with the Mascot search engine (a2). (**b**) RNA pull-down assay was performed (b1) and the associated proteins were detected with anti-EGFR antibody (b2). A schematic map of potential EGFR binding regions (R1 to 3) in lnc-EGFR (b3). Triangles indicate deletion mutations. (**c**) Lnc-EGFR specifically interacts with EGFR. c1: RIP assays were performed using CD4^+^ T cells transduced with either lnc-EGFR or lnc-EGFRΔR1 lentiviral particles, and anti-EGFR or anti-PDGFR antibodies. The precipitated RNAs were determined by qPCR for lnc-EGFR, lnc-EGFRΔR1, GAPDH or U6. c2: CD4^+^ T cells were transduced with different doses of lnc-EGFR lentiviral particles and the association of lnc-EGFR with EGFR was determined by RIP assay using anti-EGFR antibodies and qPCR for lnc-EGFR. c3: The amplified sequence (Lnc-EGFR range from 337 to 379 bp) was validated by Sanger sequencing. Each experiment was performed triplicated. Cytoplasmic lnc-EGFR bind specifically to EGFR. (**a**) Lnc-EGFR RNA pull-down assay was performed. The associated proteins were processed and subjected to Mass Spec. followed by analysis via the Proteome Discoverer program (upper) and the NCBI protein database with the Mascot search engine (lower). (**b**) RNA pull-down assay was performed (upper) and the associated proteins were detected with anti-EGFR antibody (lower panel). (**c**) A schematic map of potential EGFR binding regions (R1 to 3) in lnc-EGFR. Triangles indicate deletion mutations. (**d**) Lnc-EGFR specifically interacts with EGFR. RIP assays were performed using CD4^+^ T cells transduced with either lnc-EGFR or lnc-EGFRΔR1 lentiviral particles, and anti-EGFR or anti-PDGFR antibodies. The precipitated RNAs were determined by qPCR for lnc-EGFR, lnc-EGFRΔR1, GAPDH or U6. (**e**) CD4^+^ T cells were transduced with different doses of lnc-EGFR lentiviral particles and the association of lnc-EGFR with EGFR was determined by RIP assay using anti-EGFR antibodies and qPCR for lnc-EGFR. (**f**) The amplified sequence (Lnc-EGFR range from 337 to 379 bp) was validated by Sanger sequencing. Each experiment was performed triplicated.

According to the chromosome location, lnc-EGFR is overlapped with the protein coding gene RNASE4. The specific primers and shRNAs for lnc-EGFR were designed in order to avoid the overlapping with RNASE4 (Supplementary Fig. 7). As expected, knockdown of lnc-EGFR had no apparent effect on expression of RNASE4 (Supplementary Fig. 7), suggesting that the oligo sequences for lnc-EGFR experiments are specific. We next used a pull-down assay with biotinylated lnc-EGFR to search for potential lnc-EGFR-interacting proteins. Mass spec analysis of pull-down proteins revealed that the previously predicted lnc-EGFR partner, EGFR, was a lnc-EGFR-associated protein (Fig. 3a and Supplementary Fig. 8a). Our immunoblotting analysis further confirmed this finding (Fig. 3b). On the basis of these results, the bioinformatic software catRAPID was used to predict the potential EGFR binding regions in lnc-EGFR and the potential protein-binding domains for lnc-EGFR in EGFR. As shown in Fig. 3b (lower panel) and Supplementary Fig. 8b,c, three regions (R1 to R3) in the lnc-EGFR were predicted to bind to EGFR and the amino acids between 1,001 and 1,051 in the EGFR were suggested to be the most possible lnc-EGFR binding domain. Our pull-down results showed that deletions in the R1 (lnc-EGFRΔR1-1, lnc-EGFRΔR1-2), but not in the R2 (lnc-EGFRΔR2) or R3 (lnc-EGFRΔR3), of lnc-EGFR abrogated lnc-EGFR’s binding ability to EGFR (Fig. 3b and Supplementary Fig. 8d). RNA immunoprecipitation (RIP) assays revealed that anti-EGFR, but not anti-PDGFR, antibodies specifically precipitated lnc-EGFR, but not lnc-EGFRΔR1, GAPDH or U6 RNAs (Fig. 3c, upper panel). Further dose response analysis indicated that the association of lnc-EGFR with EGFR was in a dose-dependent manner (Fig. 3c, lower panel). Taken together, our results suggest that lnc-EGFR specifically interacts with EGFR and that the R1 is important for lnc-EGFR to bind to EGFR.

**Figure 4:**
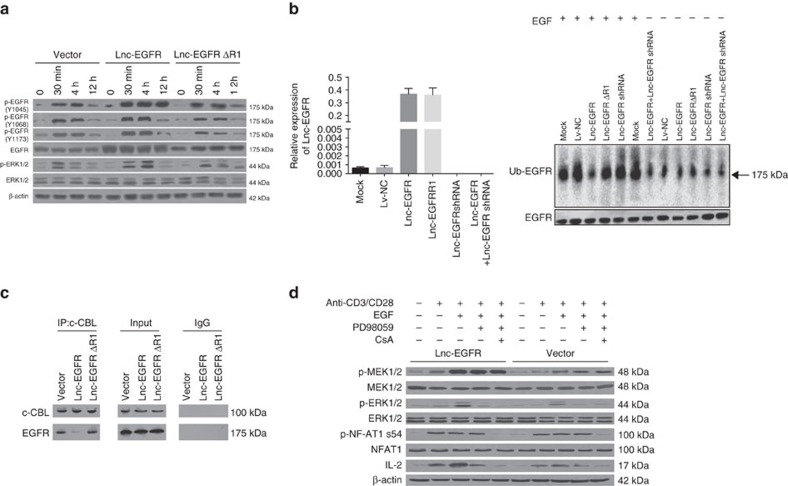
Lnc-EGFR prevents the ubiquitination of EGFR by binding to Tyr1045. (**a**) T cells isolated from peripheral blood of HCC patients were transduced with indicated lentiviral particles and then treated with with EGF (20 ng ml^−1^) for indicated timepoints followed by western blotting for p-EGFR(Y1045), p-EGFR(Y1068), p-EGFR(Y1073), p-ERK1/2(T202/Y204), EGFR, ERK1/2 and β-actin. (**b**) Normal, healthy human T cells transduced with mock or indicated lentiviral particles were determined with real-time PCR (upper) and were further treated with EGF (100 ng ml^−1^) for 90 min or left untreated. Whole-cell lysates were prepared and EGFR was immunoprecipitated followed by western blotting for ubiquitin. Equal loading of EGFR was determined by western blotting via anti-EGFR antibodies (lower). (**c**) Whole-cells lysates were prepared and c-CBL was immunoprecipitated via anti-c-CBL antibody. The presence of EGFR in the immunecomplex was determined by western blotting via anti-EGFR antibody. (**d**) Transduced T cells were treated with anti-CD3/anti-CD28 beads (bead-to-cell ratio of 1:1), EGF (20 ng ml^−1^) or left untreated in the presence or absence of PD98059 (40 μM) and/or CsA (1 μM). Whole-cell lysates were prepared and subjected to Western blotting for p-ERK1/2(T202/Y204), p-MEK1/2(S217/221), p-NF-AT1(S54), ERK1/2, IL-2, MEK1/2 and β-actin. Each experiment was performed triplicated.

The direct binding of lnc-EGFR to the phosphorylation site of EGFR led us to determine if lnc-EGFR regulates the phosphorylation of EGFR. The time-dependent activation of EGFR was investigated in conventional T cells after they were treated with epidermal growth factor (EGF) for various periods. Lnc-EGFR, but not lnc-EGFRΔR1, lnc-EGFRΔR2 or lnc-EGFRΔR3, strikingly induced expression of EGFR, increased and sustained its phosphorylation at tyrosine (Y) 1045, Y1068 and Y1173 residues in response to EGF (Fig. 4a and Supplementary Fig. 9a,b).

The domain (1,001–1,051 amino acids) of EGFR is exposed in the cytoplasm; the phosphorylation of Y1045 creates a major docking site for the ubiquitin ligase, casitas B-lineage lymphoma (c-CBL), leading to EGFR ubiquitination and the subsequent degradation^28^,^29^. We next determined the effect of lnc-EGFR on the ubiquitination and stability of EGFR. Overexpression of lnc-EGFR, but not lnc-EGFRΔR1, in normal, healthy CD4^+^ T cells strikingly diminished EGFR ubiquitination in response to EGF; knockdown of lnc-EGFR expression by co-transduction of its shRNA largely impaired its inhibitory effect on EGFR ubiquitination (Fig. 4b). Interestingly, overexpression of lnc-EGFR in HCC patient CD4^+^ T cells had a minimal effect on EGFR ubiquitination (Supplementary Fig. 10a,b). This was caused by about six times higher basal level of lnc-EGFR in HCC patient CD4^+^ T cells than normal healthy controls; knockdown of the endogenous lnc-EGFR by its shRNA restored EGFR ubquitination in response to EGF (Fig. 4b and Supplementary Fig. 10a,b). These results suggest that when lnc-EGFR reaches a certain level, it is sufficient to inhibit EGFR ubiqutination in response to EGF. Moreover, it seemed that overexpression of lnc-EGFRΔR1 interfered with the ability of endogenous lnc-EGFR to inhibit EGFR ubiquitination in HCC patient T cells in response to EGF. To elucidate the mechanism of how lnc-EGFR blocks EGFR ubiquitination, we determined if lnc-EGFR interferes with the interaction of EGFR with c-CBL by performing an immunoprecipitation assay. As shown, lnc-EGFR, but not its R1 mutant or control diminished the interaction of EGFR with c-CBL (Fig. 4c), suggesting that lnc-EGFR binds to EGFR, blocking its ubiquitination by c-CBL and the subsequent degradation.

To determine which pathways downstream of EGFR involve lnc-EGFR signalling, we first examined the PTEN/phosphoinositide 3-kinase (PI3K) axis, which regulates Treg differentiation. We found that overexpression of lnc-EGFR had an apparent effect on PI3K activation and PTEN expression (Supplementary Fig. 11). Then, we determined if both the EGFR/RAS/MEK/AP1 and calcineurin/NF-AT1 pathways, which also regulate Tregs, are altered in the presence of lnc-EGFR. When CD4^+^ T cells transduced with either the control vector or lnc-EGFR were treated with anti-CD3/anti-CD28 beads, the phosphorylation of MEK1/2, ERK1/2 and NF-AT1 was increased (Fig. 4d). Interleukin 2 (IL-2), which is a classic downstream target of NF-AT1, was induced by overexpression of lnc-EGFR and further enhanced in the presence of EGF, all of which were diminished by ERK1/2 or NF-AT1 inhibitors (Fig. 4d), suggesting that ERK1/2 and NF-AT1 are effectors of lnc-EGFR and EGF.

### NF-AT1/AP1 enhances lnc-EGFR and *EGFR* in CD4^+^ T cells

**Figure 5:**
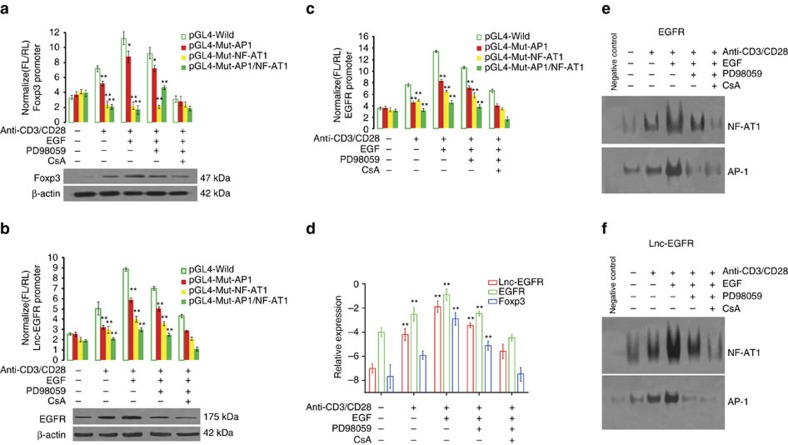
NF-AT1/AP1 complex enhances the transcription of *Foxp3* lnc-EGFR and *EGFR* in conventional CD4^+^ T cells. (**a**–**d**) The binding sites for AP1 and/or NF-AT1 in the *Foxp3* (**a**), *EGFR* (**b**), and lnc-EGFR (**c**) promoter regions were mutated and then cloned into the pGL4 vector; the empty vector was used as the control. CD4^+^ T cells were treated with TGF-β (2 ng ml^−1^), anti-CD3/anti-CD28 beads (bead-to-cell ratio of 1:1), EGF (20 ng ml^−1^), PD98059 (40 μM), and /or CsA(1 μM), and their fluorescence intensity (FL) measured by comparison with the intensity of *Renilla* fluorescence(*n*=6 for each group, Student’s *t*-test). Expression of Foxp3 protein was confirmed with western blotting in the lower panel and its mRNA expression was confirmed with RT–PCR (**d**) (*n*=6 for each group, Student’s *t*-test). (**e**,**f**) Binding of specific nuclear factors was demonstrated with EMSA analysis of the promoter region of *EGFR*; f panel indicates lnc-EGFR. Competition experiments were performed by preincubating nuclear extracts with AP1/NF-AT1 oligonucleotides by treating with anti-CD3/anti-CD28 beads (bead-to-cell ratio of 1:1) and EGF (2 ng ml^−1^) in the presence or absence of PD98059 (40 μM) and/or CsA (1 μM). Each experiment was performed triplicated. Data are presented as means±s.e.m. (*P<0.05, **P<0.01).

A bioinformatics-based prediction of transcription factors indicated that potential NF-AT1- and AP1-binding sequences are present within the promoters of *Foxp3*, lnc-EGFR, and *EGFR*. Detailed screening with a dual-luciferase reporter assay based on these predictions suggested that *EGFR* (nt –849 to –857) and *EGFR* (nt –655 to –663) are binding sites for NF-AT1 and AP1, respectively, whereas lnc-EGFR (nt –127 to –135) and lnc-EGFR (nt –90 to –98) are binding sites for NF-AT1 and AP1, respectively (Supplementary Fig. 12). The binding sites for NF-AT1 and AP1 in the promoter of *Foxp3* have been identified previously^30^. The three promoters were cloned and used as controls. The binding sequence of each transcription factor in the NF-AT1/AP1 complex was mutated. All the promoter sequences were subcloned into the pGL4 plasmid and used to transfect CD4^+^ T cells, and the promoter activities were monitored. After treatment with TGF-β, the promoter activities of all three genes increased significantly when the cells were treated with anti-CD3/anti-CD28 beads, and increased even more when they were treated with EGF. These increases were attenuated by either a site-specific mutation in AP-1 or the ERK1/2 inhibitor, PD98059 (Fig. 5a–c). The promoter activities decreased even more dramatically than the dissociation with AP1 when either the binding site on NF-AT was mutated or calcineurin/NF-AT1 signalling was blocked by its inhibitor, CsA (Fig. 5a–c). The lnc-EGFR, EGFR mRNA and Foxp3 mRNA levels in CD4^+^ T cells during various treatments were determined with real-time PCR, and were consistent with their promoter activities. The transcription of lnc-EGFR, *EGFR*, and *Foxp3* increased when NF-AT1 was activated by anti-CD3/anti-CD28 beads, and increased even further with EGF treatment. However, their transcription decreased when the activation of ERK1/2 was blocked or when NF-AT1 activation was blocked by CsA (Fig. 5d). The protein levels of both EGFR and Foxp3 were consistent with their transcript levels, which suggested that the transcription of lnc-EGFR, *EGFR* and *Foxp3* is driven by NF-AT1 in CD4^+^ T cells treated with TGF-β, and is further enhanced by the activation of the EGFR/RAS/ERK1/2/AP1 cascade.

To confirm the findings described above, an electrophoretic mobility shift assay (EMSA) was performed to gain direct insight into the binding of transcription factors to specific promoter regions. With TGF-β treatment, anti-CD3/anti-CD28 beads dramatically increased the binding of NF-AT1 and AP1 to the promoter regions of lnc-EGFR and *EGFR*, and this binding was further reinforced by EGF. When an ERK1/2 inhibitor was added, the binding of AP1 was clearly attenuated, as was that of NF-AT1. However, the binding of NF-AT1 was almost totally abolished by CsA, which had almost no effect on AP1 binding (Fig. 5e,f). Our data suggest that NF-AT1 and AP1 are transcription factors for *Foxp3*, lnc-EGFR, and *EGFR* during Treg-cell differentiation, and that NF-AT1 is the dominant factor in the transcription of these RNAs, whereas AP1 reinforces their transcription.

### Lnc-EGFR promotes Treg and inhibits CTL activity *in vitro*

**Figure 6:**
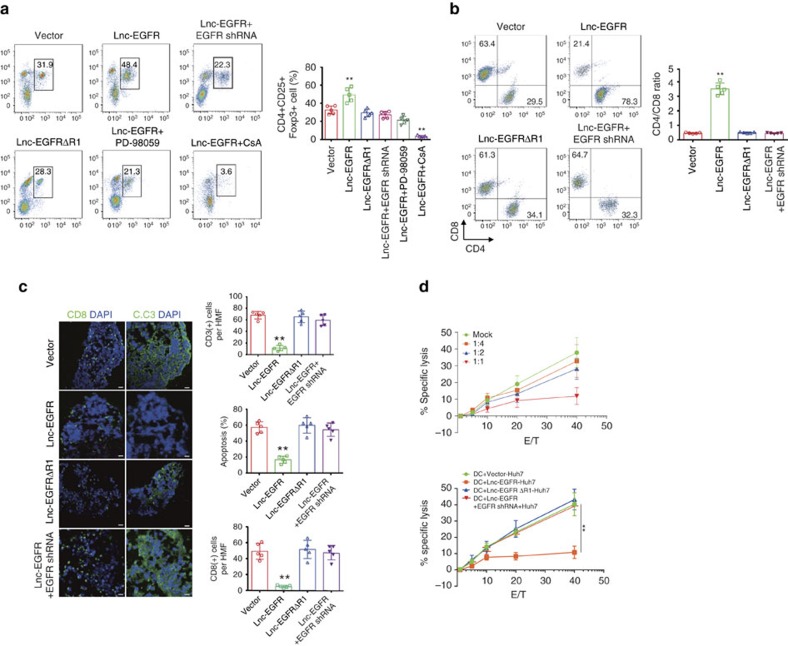
Lnc-EGFR promotes iTreg differentiation and inhibits CTL activity *in vitro*. (**a**) Different percentages of Tregs in T cells transduced with lnc-EGFR alone or combined with EGFR shRNA lentiviral particles in the presence or absence of PD98059 (40 μM), or CsA(1 μM), or transduced with lnc-EGFRΔR1 lentivira particles in a Treg cell polarization stimulation assay (with 2 ng ml^−1^ TGF-β). Quantitative results are shown on the right (*n*=6 for each group, Student’s *t*-test). (**b**) CTL suppression assay of lnc-EGFR was performed in a mixed culture of various types of CD4^+^ cells, CD8^+^ cells and OVA-induced DCs. The initial ratio of CD4^+^ cells to CD8^+^ cells was 1:1. The CD4:CD8 ratios are presented in the right panel(*n*=6 for each group, Student’s *t*-test). (**c**) A 3D culture system was used to simulate the tumour microenvironment, composed of a human HCC cell line (97H cells), DCs vaccinated with 97H cell lysate, various CD4^+^ cells, and CD8^+^ cells, evaluated with immunofluorescent staining for C.C3. The green stain in the right panel indicates cleaved caspase 3, whereas the green stain in the left panel indicates CD8^+^ cells. DAPI was used to stain the nuclei. The calculated data are given in the right panel(*n*=6 for each group, Student’s *t*-test). (**d**) The cytolytic activity of the generated CTLs based on different ratios of CD4^+^ cells was determined in a standard ^51^Cr-release assay with E: T ratios of 5:1, 10:1, 20:1 and 40:1. The cytolytic activity of the generated CTLs co-cultured with CD4^+^ cell treated with different groups was detected with a ^51^Cr-release assay and is presented in the right panel are means±s.e.m. (*n*=5 each group) (*P<0.05, **P<0.01). Each experiment was performed triplicated.

Tregs generally suppress or downregulate the induction and proliferation of effector T cells, which constitute the major part of the immunosuppressive activity within the tumour microenvironment. Therefore, a series of *in vitro* experiments were performed to investigate the immunosuppressive effects of lnc-EGFR in Tregs. First, a polarization stimulation assay was performed. After stimulation with TGF-β and anti-CD3/anti-CD28 beads for 7 days, the percentage of Tregs (CD4^+^CD25^+^Foxp3^+^) increased significantly when lnc-EGFR was overexpressed, but was restored to normal levels by EGFR knockdown (Fig. 6a and Supplementary Fig. 13). Moreover, the percentage of Tregs decreased when either the R1 in the lnc-EGFR was deleted or ERK1/2 was blocked, but was substantially decreased when NF-AT1 was blocked (Fig. 6a and Supplementary Fig. 13). Interestingly, knockdown of lnc-EGFR had no apparent effect on the basal level of Tregs during a normal differentiation process (Supplementary Fig. 14), indicating that the basal level of lnc-EGFR is low and its effect at this level on Treg differentiation is minimal.

A CTL suppression assay of lnc-EGFR was performed with a mixed culture of various types of CD4^+^ T cells, CD8^+^ T cells and ovalbumin (OVA)-induced DCs. The initial ratio of CD4^+^ and CD8^+^ cells was 1:1. Three days after mixed culture, this ratio was decreased in the vector-transduced or lnc-EGFRΔR1-transduced groups, reflecting the strong proliferation of CTL cells after OVA stimulation. However, CTL proliferation was significantly blocked by CD4^+^ cells transduced with lncRNA-EGFR, and was restored by EGFR knockdown in CD4^+^ T cells (Fig. 6b). A three-dimensional (3D) culture system was used to simulate the tumour microenvironment affected by lnc-EGFR. The 3D culture system was composed of the human HCC cell line (97H cells), DCs vaccinated against the 97H cell lysate, various CD4^+^ cells and CD8^+^ cells. After 5 days in co-culture, the apoptosis of the 97H cells was evaluated using immunofluorescent staining with anti-cleaved caspase 3 (C.C3) antibodies or propidium iodide (PI)–annexin V. The CD4^+^ cells transduced with lnc-EGFR dramatically attenuated the antitumor effect exerted by CD8^+^ cells. The C.C3 level was increased in 97H cells co-cultured with CD4^+^ cells if lnc-EGFRΔR1 was overexpressed or EGFR was knocked down regardless of the presence or absence of lnc-EGFR (Supplementary Fig. 15). This result further suggests that lnc-EGFR-mediated effects of Tregs are EGFR-dependent.

The percentage of CD8^+^ cells was also assessed via immunofluorescent staining and flow cytometry. The proportion of CD8^+^ cells decreased when co-cultured with lnc-EGFR-transduced CD4^+^ cells and was rescued by EGFR knockdown (Fig. 6c). Moreover, a ^51^Cr-release assay was performed to investigate the suppressive effect of lnc-EGFR on CD4^+^ T cells. An HCC cell line, Huh7, was labelled with ^51^Cr and then co-cultured with Huh7-vaccinated DCs and various CD4^+^ T cells, treated as described above. Reduced absorbance was observed in the group co-cultured with transduced CD4^+^ T cells, indicating the stronger immunosuppressive effect conveyed by lnc-EGFR. This immunosuppression was attenuated by CD4^+^ T cells transduced with lnc-EGFRΔR1 or EGFR shRNA lentivirus (Fig. 6d).

### Lnc-EGFR enhances tumour growth *in vivo*

**Figure 7:**
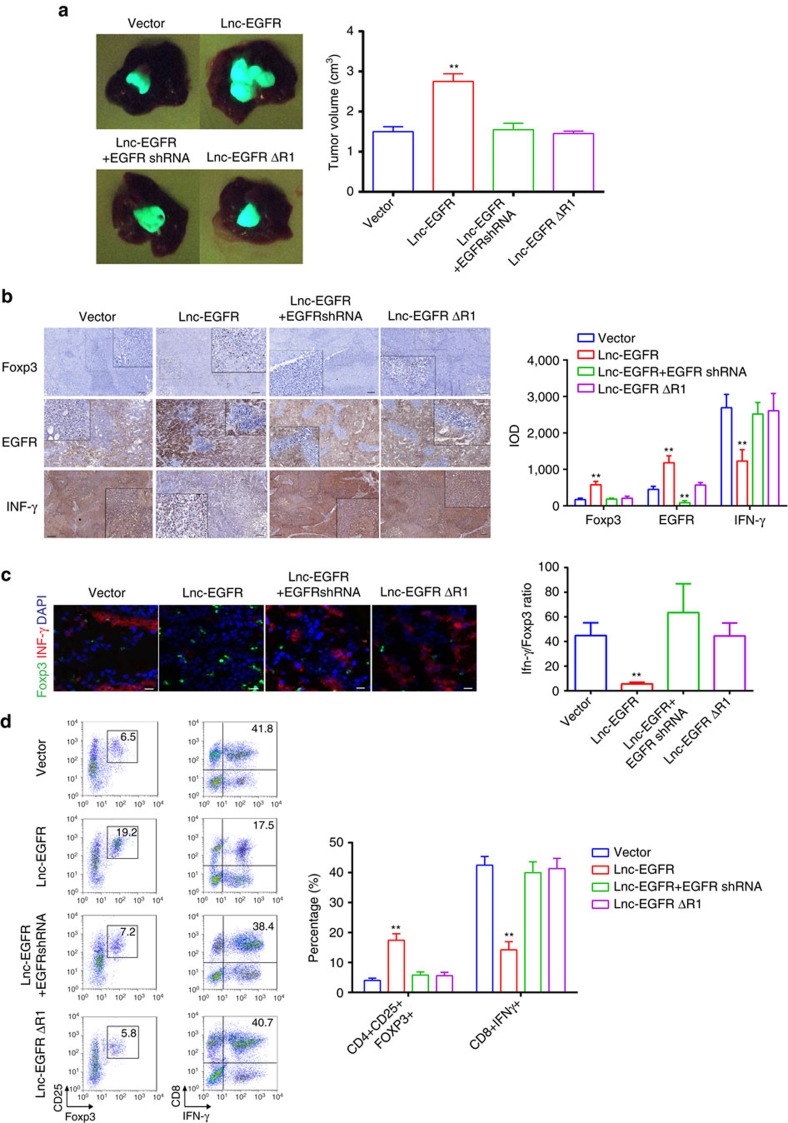
Lnc-EGFR enhances tumour growth *in vivo* by promoting Treg differentiation. (**a**) Tumour growth *in vivo* by orthotopic transplantation with adoptive cell transfer using CD4^+^ T cells transduced with lentiviral particles overexpressing indicated vectors. Shown are representative images of tumours in the liver (left panel). Tumour volumes within different groups of the experiment (right panel). (**b**) Immunohistochemistry was performed in paraffin sections of the tumours for Foxp3, EGFR and IFN-γ, and the integrated optical density was calculated as mean value of five random views (× 100). (**c**) Foxp3 and IFN-γ were detected in frozen sections of the tumour tissues; the red staining indicates Foxp3 and the green staining indicates IFN-γ. DAPI was used to stain the nuclei (× 200). Quantitative results are listed on the right, as means±s.e.m. (*n*=6 for each group, Student’s *t*-test) (**d**) The distributions of Tregs and CTLs in T cells isolated from the tumours were determined by flow cytometry (*n*=6 for each group, Student’s *t*-test). Each experiment was performed triplicated. Data are presented as means±s.e.m. (*P<0.05, **P<0.01).

The biological effect of lnc-EGFR was further elucidated *in vivo* by orthotopic or subcutaneous tumour transplantation with adoptive cell transfer in non-obese diabetic/severe combined immune deficiency (NOD/SCID) mice using CD4^+^ T cells transduced with the control vector, lnc-EGFR, lnc-EGFR+EGFRshRNA or lnc-EGFRΔR1, and 97H-cell-vaccinated DCs. The time-based overexpression or knockdown were confirmed (Supplementary Fig. 16). Tumour growth was dramatically suppressed by the tumour-cell-vaccinated DCs; however, this antitumor effect was strongly attenuated by the cooperation of lnc-EGFR-infected CD4^+^ T cells, but was rescued by EGFR knockdown or lnc-EGFRΔR1 (Fig. 7a and Supplementary Fig. 17a). The tumour tissues were co-stained for Foxp3 and IFN-γ and the Foxp3/IFN-γ ratio was calculated. IFN-γ^+^ cells decreased, whereas Foxp3^+^ cells increased in the tumours composed of lnc-EGFR, but not lnc-EGFRΔR1, -transduced CD4^+^ T cells. This effect of lnc- was reversed when EGFR was knocked down (Fig. 7b and Supplementary Fig. 17b). Similar results were obtained with immunohistochemical staining: the percentage of Foxp3 and the expression of EGFR were significantly higher in the infiltrating T lymphocytes in the lnc-EGFR-transfected group than in the tumour tissues containing the vector-, Lnc-EGFR+EGFRshRNA or lnc-EGFRΔR1-transduced CD4^+^ cells; IFN-γ decreased dramatically when lnc-EGFR-transduced CD4^+^ cells were present. (Fig. 7c and Supplementary Fig. 17c). Finally, the percentages of Tregs and CTLs were analysed with flow cytometry. The percentage of Tregs increased, whereas the percentage of CTLs (CD8^+^IFNγ^+^) decreased in the lnc-EGFR group compared with those in the other three groups. There was no significant difference in the percentages of Tregs and CTLs among vector, Lnc-EGFR+EGFR shRNA or lnc-EGFRΔR1 (Fig. 7d and Supplementary Fig. 17d).

## Discussion

TILs are a type of leukocytes found in tumours. TILs were previously thought to be capable of eliminating tumour cells, and studies demonstrated that the presence of lymphocytes in tumours is often associated with better clinical outcomes^31^,^32^. However, a large mountain of evidence has demonstrated that TILs including Tregs trigger chronic inflammation, which is one of several key risk factors for tumorigenesis, invasion and metastasis, and has therefore become an enabling characteristic of cancer^33^. TILs are mainly derived from the peripheral blood, and upon infiltration into the tumour site their functions change greatly, including their differentiation stages, surface markers, secretion profiles and so on^32^,^34^. We hypothesized that the functional switch of TILs is induced by the change in their expression profiles. Therefore, the expression profiles of both mRNAs and lnc-RNAs in CD4^+^ T cells isolated from HCC tissues and the PBLs of HCC patients and healthy controls were analysed, and a lnc-RNA/mRNA network was built up and discovered that there is a forward-feedback loop, lnc-EGFR-EGFR-NF-AT1/AP1-lnc-EGFR, in Tregs in HCC.

Malignant tumours that grow and progress are the ones that successfully avoid immune destruction, which is also defined as immunosuppression-related immune escape^35^. Different escape mechanisms can bypass immune surveillance, including immune editing and the induction of tolerance. The elevated expression of immunosuppressive molecules, which trigger various immune checkpoints, is mainly responsible for the establishment of immune tolerance in cancers. However, there are additional tolerogenic mechanisms, including the apoptotic deletion of immune effector cells by death-inducing ligands, the toleration of tumour-reactive T cells by immunosuppressive cytokines, such as TGF-β, the suppression of immune-reactive T cells by Tregs, and polarization by antigen-cross-presenting cells, such as DCs^36^,^37^. Among these factors, Tregs have most frequently been studied in both clinical samples and animal models. Tregs are a component of the immune system that suppresses the immune responses of other cells, and are defined as CD4^+^CD25^+^Foxp3^+^ T cells. Tregs have been considered as a therapeutic target of chemotherapy^38^,^39^.

LncRNAs are novel subsets of noncoding RNAs, which regulate a variety of biological responses via a diverse range of mechanisms. Recent studies have shown that numerous changes in the expression of lncRNAs occur during the activation of the innate immune response and T-cell development, differentiation, and activation^40^,^41^. We have demonstrated that Tregs in HCC and other human tumour tissues are increased upon the expression of lnc-EGFR, suggesting that the lnc-EGFR-Treg axis is a common pathway during the pathogenic process of tumorigenesis. lnc-EGFR binds to EGFR, stabilizes it and sustains activation of it and its downstream RAS/ERK/AP1 signalling, leading to Treg differentiation, CTL inhibition and HCC development.

EGFR is an important member of the receptor tyrosine kinase family. EGFR is activated by its ligands and then triggers activation of the Ras/Raf/MEK/ERK1/2 signalling pathway, which plays a critical role in cancer development. EGFR is regulated by its ubiquitination through interaction with c-CBL. Blockade of this interaction stabilizes EGFR and sustains its activation. Indeed, we have uncovered that lnc-EGFR binds to EGFR using its R1 domain, and this binding is very specific and inhibits the interaction of EGFR with c-CBL thus stabilizing EGFR and sustaining its activity. We have demonstrated that high basal levels of lnc-EGFR in HCC patients are sufficient to diminish EGFR ubiquitination, thereby expanding its lifespan and enduring its activation. Our study provides a rationale for increased levels of EGFR and its persistent activation as well as Tregs observed in HCC patients. Our study is the first evidence showing that, in addition to protein factors, EGFR stability and activation are regulated by nuclear acids.

EGFR plays an important role in immune cell regulation in the tumour microenvironment, and has been a therapeutic target for many cancers. For example, cetuximab, a specific EGFR inhibitor, has been used for the treatment of metastatic colorectal cancer, metastatic non-small cell lung cancer and head and neck cancer. Cetuximab therapy is accomplished through increasing tumour-specific CTL activity and reducing Tregs^42^, thus revealing a link between EGFR and Tregs. Recently, a study suggested that the activation of EGFR by its ligand, amphiregulin (AREG), enhances the function of Tregs in a colitis and tumour vaccination model^43^. Our previously study suggested that EGF and AREG are abundant in human liver cancer. Now we demonstrate that these ligands not only activate EGFR and NF-AT1/AP-1 in Tregs but also stimulate expression of EGFR, Foxp3 and lnc-EGFR.

NF-AT transcription factors induce the expression of genes important for the development and activation of lymphocytes. They are expressed in a variety of leukocytes, including T cells, B cells, NK cells, and monocytes, and nonimmune-related cells (cardiac, muscle and neuronal cells)^44^. The NF-AT proteins interact with different transcription factor partners in the nucleus, and are important integrators of calcium signalling and many other signalling pathways in T cells. AP1 proteins are the main transcription partners of NF-AT1 during T-cell activation. Dimers of FOS and JUN form quaternary complexes with NF-AT1 and DNA on NF-AT1/AP1 composite sites, which contain two adjacent binding motifs for both transcription factors and are present in many genes induced during T-cell activation^45^,^46^. A study by Mantel *et al*. indicated that the basal promoter contains six NF-AT1 and AP-1 binding sites, which positively regulate the *trans* activation of the *Foxp3* promoter after the T-cell receptor is triggered^43^. In the present study, we have found that NF-AT1/AP1-composite-binding sites exist in all the promoters of lnc-EGFR, EGFR and Foxp3 genes, and their expression is greatly enhanced by the activated calcineurin/NF-AT1 and EGFR/AP1 cascades.

**Figure 8:**
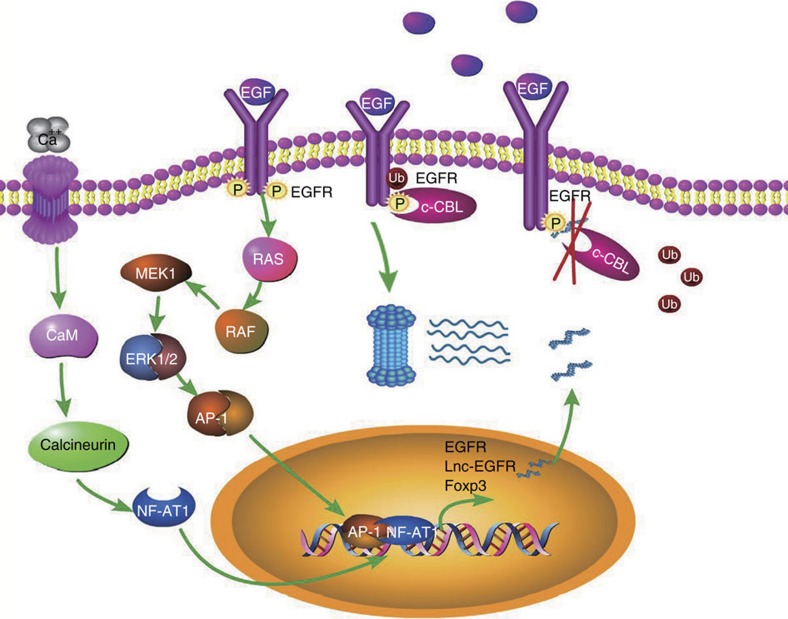
Schematic model of the forward-feedback loop. Lnc-EGFR specifically binds to EGFR, stabilizes it through blocking its interaction with c-CBL and the subsequent ubiquitination, and sustains its activity, leading to subsequent downstream cascade activation, Treg differentiation, CTL inhibition and HCC progression.

In summary, we have discovered a novel lncRNA, lnc-EGFR, which links Tregs and HCC. We have demonstrated that lnc-EGFR specifically binds to EGFR, stabilizes it through blocking its interaction with c-CBL and the subsequent ubiquitination, and sustains its activity, leading to subsequent downstream cascade activation, Treg differentiation, CTL inhibition and HCC progression (Fig. 8). Moreover, we have demonstrated the presence of a forward-feedback loop in the Tregs, in which lnc-EGFR activates EGFR, which in turn activates ERK1/2 and AP-1, triggering AP1-dependent lnc-EGFR and Foxp3 expression. Given that Treg cells are closely associated with HCC progression^47^,^48^, lnc-EGFR stimulates Treg expansion through EGFR, and the inhibition of EGFR increases the anti-HCC tumour activity of sorafenib^49^,^50^, the only drug approved for HCC therapy, we speculate that the breakdown of this forward-feedback loop by managing lnc-EGFR may improve the efficacy of sorafenib in the treatment of advanced HCC.

## Methods

### Clinical samples

Blood or tissue samples from 55 healthy volunteers and 70 HCC patients who received treatment between August 2013 and May 2015 at The First Affiliated Hospital of Nanjing Medical University (Nanjing, Jiangsu, China) were used for isolation of peripheral or tissue-infiltration lymphocytes, or for immunostaining. None of the patients had received anticancer therapy before surgery, and individuals with concurrent autoimmune disease, HIV, or syphilis were excluded. Clinical characteristics were classified according to the guidelines of Union for International Cancer Control (UICC TNM). All experiments were performed in compliance with government policies and the Helsinki Declaration. The individuals were informed about the study and gave consent prior to the specimen collection. And the research has been approved by an ethics committee of the First Affiliated Hospital of Nanjing Medical University.

### Cell culture

Human HCC cell lines (97H and Huh7) were maintained in Dulbecco’s modified Eagle’s medium (Invitrogen Life Technologies, CA, USA) supplemented with 10% fetal bovine serum (FBS, Gibco, CA, USA). 97H was purchased from cell bank of Chinese Science Academy. Huh7 and 293 T cells obtained from ATCC were cultured in DMEM supplemented with 10% FBS. All cell lines were tested for mycoplasma contamination.

### Mutagenesis of Lnc-EGFR and Lentiviral packaging

The full-length and mutants of Lnc-EGFR were synthesized by Genscript Co. Ltd. (Nanjing, China) based on the sequence indicated in Supplementary Fig. 8b. Then the sequences were subcloned into pLV-His or pLV-Luc plasmid, and further packaged for lentivrial particles according to the method as previously described^5^ In brief, candidate plasmid was co-transfected with VSV-G and dR8.91 in 293 T cell line. The supernatant was collected after culturing for 72 h. Virus supernatant was concentrated through ultracentrifugation.

### Lymphocyte Isolation And HCC-specific DCs generation

PBLs were isolated by Ficoll (BD Pharmingen, CA, USA) density gradient centrifugation^51^. Fresh TILs were obtained as described previously^52^. Briefly, liver cancer tissue specimens were cut into small pieces and digested in RPMI 1640. Dissociated cells were filtered through a 75 μm cell strainer and separated by Ficoll centrifugation, and the mononuclear cells were washed and resuspended in RPMI 1640 supplemented with 10% FBS (Gibco). T cells were purified with anti-CD3 magnetic Dynabeads (Invitrogen Life Technologies) according to manufacturer’s instruction. Both the mutation and wild type sequences were inserted into the lentiviral vector –pLV (Clontech, CA, USA) to generate expression vectors. These expression vectors were mixed with lentiviral packaging Δ8.91 and envelope expressing VSV-G plasmids to generate lentiviral particles in 293 T cells. Viral particles were concentrated by ultracentrifugation and expression vector titres were determined. The CD4^+^ T cells isolated from HCC patients were cultured with TAKARA GT-T551 medium supplied with human IL-2, and then transduced with lentivirus with desired expression vectors.

For generation of HCC-specific DCs, the peripheral blood mononuclear cells (PBMCs) were isolated by centrifugation over Ficoll. Enrichment of monocytes was performed by negative selection by using immunomagnetic beads. GM-CSF (50 ng ml^−l^; R&D Systems, Minneapolis, MN, USA) and IL-4 (50 ng ml^−1^; R&D Systems) was added to generate DCs. After 5 days of culture, cells were immunized with cell whole lysate of 97H cells for 48 h, leading to fully mature DCs.

### RNA isolation and quantitative reverse transcription PCR (RT–PCR)

Total RNA was isolated with Trizol and purified with the RNeasy MinElute Clean up kit (Qiagen, Hilden, Germany) according to the manufacturer’s instruction. The cDNA was synthesized from the total RNA using the random priming method^53^. Transcript levels were measured in duplicate by quantitative reverse transcription PCR (ABI 7900; Life Technologies). Expression levels were calculated relative to GAPDH. Primer pairs used in SYBR Green reactions are listed in Supplementary Table 1.

### Microarray detection and screening workflow

Total RNA was isolated from 1 × 10^6^ T cells and used for the lncRNA/mRNA integrated microarray analysis (Capitalbio, Beijing, China). The sample preparation and microarray hybridization were performed according to the manufacturer’s instruction with minor modifications. Briefly, mRNA was purified from total RNA after removal of rRNA (mRNA-ONLY Eukaryotic mRNA Isolation Kit, Epicentre, WI, USA), amplified and transcribed into fluorescent cRNA along the entire length of the transcripts without 3′ bias utilizing a random priming method. The arrays were scanned by the Agilent Scanner (Agilent, CA, USA). The detailed information of the microarray was submitted to ArrayExpress (accession code E-MTAB-3553).

Agilent Feature Extraction software (version 11.0.1.1) was used to analyse acquired array images. Quantile normalization and subsequent data processing were performed using the GeneSpring GX v12.0 software package (Agilent Technologies). After quartile normalization of the raw data, lncRNAs and mRNAs, which had flags in Present or Marginal (All Targets Value) in at least six out of nine samples were chosen for further analysis. LncRNA and mRNA expression patterns were revealed via Hierarchical Clustering. Pathway analysis was performed via KEGG, Biocarta and Reatome software. The Fisher’s exact test and *χ*^2^-test were used to identify the significant pathways. The threshold of significance was defined by *P* value and false discovery rate (FDR). The mRNA expression in the candidate pathway was validated by RT–PCR and lncRNA/mRNA co-expression network was built to identify the correlated lncRNAs, which were subsequently validated by RT–PCR. The screening workflow chart was depicted in Supplementary Fig. 1. The detailed probe number and ENST name was presented in Supplementary Table 2.

### Co-expression network and feed-forward loop prediction

For the co-expression network, the system was built according to the normalized signal intensity of expression of specific genes and lncRNAs. For each pair of mRNA-lncRNA or mRNA-mRNA, Pearson Correlation was employed and the significant correlation pairs were used to construct the network. When networks were sampled the degree centrality became the simplest and most important measure of a gene or lncRNA centrality within a network. Moreover, the Network Structure Analysis was carried out to locate core regulatory factors, which connected most adjacent mRNAs and lncRNA, and were determined by the degree differences between two class samples. Cytoscape (Gladstone Institutes, CA, USA) was applied for the presentation of predicted network.

Based on the co-expression network, the candidate transcription factors were predicted via Transcription factor Analysis (TFscan). First, the sequences of differential expression genes were identified, and their relationship with transcription factors was determined via the Jemboss software. Next, a transcription factor regulation network (TF-mRNA-Network) was built to predict the interactions of gene promoters with transcription factors. Pearson Correlation was used to analyse the correlations between transcription factors and their target genes and mRNAs.

### *In Situ* Hybridization (ISH)

*In Situ* Hybridization was performed by employing the ISH kit from Boster (Wuhan, China) as previously described^54^. Cells in the clinical specimens(10 μm) were fixed and permeablized using xylenes, ethanol and protease to allow biotin-labelled probes to access. Slides were treated with 30% H_2_O_2_ and ddH_2_O with the ratio of 1:10 for 5 min, and then the 3% citric acid diluted pepsase was applied to expose the fragment of nucleic acid for 20 s.The second fixation was followed by using 1% paraformaldehyde/0.1 M PBS. Next, the slides were incubated with pre-hybridization solution at 40 °C for 2 h and then with lncRNA target probes at 30 °C overnight followed by two washes with 2 × saline sodium citrate. After blocking, biotin-labelled anti-digoxin was added and incubated for 60 min. Finally, slides were stained with DAB, dehydrated with 100% ethanol and xylene, and mounted in a xylene-based mounting media. The slides were recorded by Pannoramic SCAN (3DHISTECH, Budapest, Hungary) and analysed by Pannoramic Viewer (3D HISTECH, Budapest, Hungary).

### Immunohistochemistry and immunofluorescence

Paraffin-embedded and formalin-fixed HCC samples were used for immunohistochemistry detection as previously described^55^. Antibodies (Ab) including human EGFR (1:100, ab30), Foxp3 (1:100, ab2481), IFN-γ (1:200, ab9657), cleaved Caspase 3 (C.C3) (1:200, ab2302) and IL-10 (1:100, ab34843) (Abcam, Cambridge, UK) were used. For immunofluorescence analysis, tissue slides were stained with rabbit anti-human Foxp3, rabbit anti-human cleaved Caspase 3, mouse anti-human EGFR, and goat anti-human IFN-γ antibodies, followed by staining with Alexa Fluor 488-conjugated anti-mouse IgG (1:500, Ab150117), Alexa Fluor 555-conjugated anti-rabbit IgG(1:1,000, Ab150074), and Alexa Fluor 647-conjugated anti-Rabbit IgG (1:500, Ab150075) (Abcam, Cambridge, UK) antibodies. Positive cells were quantified using Image-Pro Plus software (Media Cybernetics, MD, USA) and detected by confocal microscopy (Zeiss, Oberkochen, Germany).

### *In vitro* stimulation of Treg cell and Flow cytometry (FCM)

Magnetic beads isolated peripheral T lymphocytes, tumour-infiltrating T lymphocytes, and various genes modified T cells were expanded with Dynabeads Human T-Activator CD3/CD28 (ThermoFisher, CA). For *in vitro* generation of Treg cell, naive T cells were isolated by human naive T-cell isolation kit II (Miltenyi, Koln, Germany), and further stimulated with human recombined human TGF-β (1 ng ml^−1^) (Peprotech, NJ) for 7 days. For detection of intracellular cytokines, T cells were stimulated at 37 °C for 5 h with Leukocyte Activation Cocktail (BD Pharmingen). Dead cells were excluded based on staining with Live/Dead fixable dye. Thereafter, cells were, fixed, permeabilized with IntraPrep reagent (BD Pharmingen), and then stained with flurochrome-conjugated labelled antibodies including CD4 (20 μl per test555346), CD8a (20 μl per test, 561949), CD25 (20 μl per test, 555432), Foxp3 (20 μl per test, 560082) and IFN-γ (20 μl per test-559327) (BD Pharmingen). Data were acquired on BD FACSVerse flow cytometer (BD Pharmingen).

### Bioinformatics analysis

The EGFR-binding site sequence was provided by the *CatRAPID* database. The full length of EGFR amino acid (Accession: NP_005219.2) and lncRNA nucleotide sequences were taken as an input. Both the TRANSFAC (www.gene-regulation.com) and PROM (http://alggen.lsi.upc.es/) were used to predict the potential binding sites for AP1 or NF-AT1 in the promoter regions of EGFR, lncRNA and Foxp3 (2,000 bp upstream flanking sequence from the TSS).

### RNA pull-down and mass spectrometry

The biotin-labelled lncRNA (both wild type and mutant type) and the antisense RNA were *in vitro* transcribed with a Biotin RNA Labelling Mix (Roche, CA, USA) and the T7 RNA polymerase (Roche), treated with RNase-free DNase I (Roche) and purified with an RNeasy Mini Kit (Qiagen). CD4^+^T cell extracts were incubated with biotinylated RNAs and 60 μl of streptavidin agarose beads (Invitrogen Life Technologies). The associated proteins were resolved by SDS–polyacrylamide gel electrophoresis, and specific bands were excised. Proteins were eluted, digested and subjected to the OrbitrapVelos Pro LC/MS system (Thermo Scientific, CA, USA). Data were analysed by Proteome Discoverer and the resulting peak lists were used for searching the NCBI protein database with the Mascot search engine.

### RNA Immunoprecipitation

RIP was carried out by using the Magna RIP RNA-Binding Protein Immunoprecipitation Kit (Millipore, MA, USA) according to the manufacturer’s instruction. Anti-EGFR and anti-PDGFR antibodies (1:30, ab30 and 1:20, ab32570; Abcam, Cambridge, UK) were used for RIP, respectively. CD4^+^ T cells were either transduced with fixed or different doses of lentivirus containing lnc-EGFR along with other indicated control virus. The co-precipitated RNAs were detected by reverse transcription PCR and quantitative PCR. The primer sequences are listed in Supplementary Table 1. Total RNAs (input controls) and IgG were assayed simultaneously to demonstrate that the detected signals were the result of RNAs specifically binding to EGFR.

### Immunoprecipitation and western blot

The whole-cell lysates were prepared as previously described^56^. Equal amounts of proteins were boiled, separated on 10% SDS–polyacrylamide gel electrophoresis, transferred onto a PVDF membrane and visualized via an ECL kit (Millipore, MA, USA). Antibodies including cCBL(1:1,000, ab32027), EGFR (1:1,000, ab2430), p-EGFR (Y1045)(1:1,000, ab24928), p-EGFR(Y1068)(1:1,000, ab32430), p-EGFR(Y1173)(1:1,000, ab5652), PDGFR (1:1,000,ab32570), MEK1/2 (1:1,000, ab178876), p-MEK1/2(1:1,000, S217/221) (1:1,000, ab194754), ERK1/2(1:1,000, ab17942), p-ERK1/2(T202/Y204) (1:1,000, ab47339) and β-actin (1:1,000, ab6276) were purchased from Abcam (Cambridge, UK)

For ubiquitination detection, whole cell lysates were prepared in a lysis buffer containing 10 mM N-Ethylmaleimide to inhibit ubiquitin conjugating enzymes. CD4^+^ T cells transduced with indicated lentiviral particles and then treated or untreated with EGF (100 ng ml^−1^) for 90 min. EGFR was immunoprecipitated, and its ubiquitination was detected with anti-ubiquitin antibody (1:1,000, ab7254) (Abcam, Cambridge, UK). For detecting interaction of EGFR with c-CBL, CD4^+^ T cells transduced with indicated lentiviral particles and then treated or untreated with EGF. Whole cell lysates were prepared and c-CBL was immunoprecipitated via a Dynabeads Protein A Immunoprecipitation Kit (Thermofisher, CA, USA) according to manufacturer’s instruction. The presence of EGFR in the immune complex was determined by Western blot using anti-EGFR antibodies. Uncropped scans of blots were supplied as Supplementary Fig. 18.

### Dual-luciferase reporter assay

CD4^+^ T cells were cultured in complete RPMI1640 medium supplemented with IL-2 (10 ng ml^−1^). The EGFR/lncRNA/Foxp3 promoter (either wild type or mutant type) luciferase reporter vectors (pGL4 packaged) and the mock pGL4 vector were electroporated into 3 × 10^6^ CD4^+^ T cells. After 24 h, cells were starved in serum-free medium and stimulated with Dynabeads Human T-Activator CD3/CD28 (ThermoFisher, CA) (Miltenyi, Koln, Germany), and luciferase activity was measured by the dual luciferase assay system (Promega, WI, USA) according to the manufacturer’s instructions. Data were normalized by the activity of Renilla luciferase.

### Electrophoretic Mobility Shift Assay (EMSA)

EMSAs were performed via a chemiluminescent EMSA kit (Invitrogen Life Technologies). Briefly, CD4^+^T cells were treated with a hypo-osmotic buffer (Sigma-Aldrich, MO, USA) containing protease inhibitors (Roche) followed by addition of NP-40 (Sigma-Aldrich) to a 1% final concentration. For supershift assays, the nuclear extract was pre-incubated with 1 μl antibody for 10 min at room temperature and incubated with probe at room temperature for 20 min. The band was visualized after treated Light Shift Substrate Equilibration Buffer and recorded by Chemi Doc MP (Bio-Rad, CA, USA).

### Standard ^51^Cr-release assays (CTL assays)

CTL assays were carried out as described previously^57^. In brief, target cells were transfected with lnc-EGFR, labelled with ^51^Cr sodium chromate in X-VIVO 20 medium for 1 h at 37 °C, and then transferred to a well of a round-bottomed 96-well plate (1 × 10^4^ per well). Varying numbers of CTLs were added and incubated for 4 h. Supernatants (50 μl per well) were collected, and the percentage of specific lysis was calculated by formula ((experimental release—spontaneous release)/(maximal release—spontaneous release) × 100%). Spontaneous and maximal release was determined in the presence of either X-VIVO 20 medium or 2% Triton X-100, respectively.

### 3D co-culture system

The primary human CD4^+^ T cells (1 × 10^6^) transduced with lentiviral particles, CD8^+^ T cells (1 × 10^6^), antigen-stimulated DCs (1 × 10^5^) stimulated with human GM-CSF and IL-4, and immunized with cell whole lysate of 97H cells and 97H cells (2 × 10^6^) were cultured in a 3D Petri Dish (Micro-Tissues) (RI, USA) according to manufacturer’s instruction. The cells were collected after 7 days, washed with PBS and then used for immune staining, cell isolation and flow cytometry analysis.

### *In vivo* model

Hepatoma cells (97H) were labelled with luciferase, and CD4^+^T cells were transduced lentiviral particles containing empty vector, WT lnc-EGFR or Lnc-EGFR-ΔR1 expression vectors. DCs (1 × 10^5^), which were stimulated by human GM-CSF and IL-4 and further immunized with the whole-cell lysate of 97H, were co-cultured with CD4^+^ T cells (1 × 10^6^), CD8^+^ T cells (1 × 10^6^) and 97H (5 × 10^6^) in 100 μl of buffered saline and were subcutaneously injected into the dorsal tissue of 5–6-week-old male NOD/SCID mice (*n*=10) which was randomized and blinded allocated into each group. Tumours were measured every week after the implantation, and the volume of each tumour was calculated (length × width^2^ × 0.5). All mice were killed 5 weeks after implantation and organs were collected for further analysis.

An orthotopic mouse model of liver cancer was carried out according to our previously report^58^. One week before transplantation, an adoptive cell transfer was performed, cell mixture containing CD4^+^ T cells (1 × 10^6^) transduced with lentiviral particles, CD8^+^ T cells (1 × 10^6^) and 97H cell vaccinated DCs (1 × 10^5^), which were mentioned above, was transplanted into the NOD/SCID mice (*n*=6) via mice tail vein. The tumour volume was calculated (length × width^2^ × 0.5). All mice were killed 5 weeks after transplantation and organs were collected for further analysis. All animal protocols have been approved by the Institutional Animal Care and Use Committee of Nanjing Medical University.

### Statistical analysis

Data are presented as mean±s.e.m. *χ*^2^-tests and the Student’s *t*-test analysis of variances were used to evaluate statistical differences in demographic and clinical characteristics. All the expression experiments we conducted *in vitro* were repeated at least three times with samples in triplicates. Pearson correlation analysis was used to analyse the relationship of associated factors. Statistical analysis was performed using STATA 9.2 and presented with the GraphPad prism software (CA, USA). In all cases, *P*<0.05 was considered significant.

### Data availability statement

The microarray data have been deposited in the ArrayExpress (https://www.ebi.ac.uk) database under the accession code E-MTAB-3553. The microarray data referenced during the study are available in a public repository from the ArrayExpress website (https://www.ebi.ac.uk). All other data all the other data supporting the findings of this study are available within the article and its Supplementary Information files or from the corresponding author upon reasonable request.

## Additional information

**How to cite this article:** Jiang, R. *et al*. The long noncoding RNA lnc-EGFR stimulates T-regulatory cells differentiation thus promoting hepatocellular carcinoma immune evasion. *Nat. Commun.* **8**, 15129 doi: 10.1038/ncomms15129 (2017).

**Publisher’s note:** Springer Nature remains neutral with regard to jurisdictional claims in published maps and institutional affiliations.

## Supplementary Material

Supplementary Information Supplementary Figures and Supplementary Tables

## Figures and Tables

**Table 1:** Relevance analysis of lnc-EGFR expression in HCC patients.

**Varible**	**lnc-EGFR**		***P*** value*
	**Low**	**High**	
All cases	26	41	
Age			0.790
<60	21	32	
≥60	5	9	
Gender			0.668
Male	22	33	
Female	4	8	
HBV			0.773
Positive	24	37	
Negative	2	4	
Differentiation grade			0.773
Well	15	20	
Moderate	7	13	
Poorly	4	8	
Tumour size(cm)			**0.002**
≤5 cm	17	11	
>5 cm	9	30	
Tumour number			0.936
Solitary	23	36	
Multiple	3	5	

EGFR, epidermal growth factor receptor; HBV, Hepatitis B virus; HCC, hepatocellular carcinoma.

^*^Data was analysed by *χ*^2^-test. *P* value in bold indicates statistically significant.
