# Supplementary material for: The long noncoding RNA lnc-EGFR stimulates T-regulatory cells differentiation thus promoting hepatocellular carcinoma immune evasion
Source: Nat Commun. 2017 May 25;8:15129. doi: 10.1038/ncomms15129 (PMC5529670; doi:10.1038/ncomms15129)
Supplement: Supplementary Information — Supplementary Figures and Supplementary Tables [file ncomms15129-s1.pdf]

Supplementary Table 1 Detailed information of oligonucleotide Sequences

Gene	Sequence	
Lnc-EGFR-1	Forward	5'- CAGCAGCCCTGCAATTAAAC -3'
	Reverse	5'- GGGTCCTCATGTAATGGTAATAGG -3'
	Probe	5'- TTCTCTGCTGCAACCCAGTGTCTT -3'
Lnc-EGFR-2	Forward	5'- AGAGCCCAACTGATGAACTG -3'
	Reverse	5'- AGGATTTGGAGTAGGTGGAAATAA -3'
	Probe	5'- TCTGTCCCATGACTTCACTCTGCA -3'
EGFR	Forward	5'-AGGCACGAGTAACAAGCTCAC-3'
	Reverse	5'-ATGAGGACATAACCAGCCACC-3'
Foxp3	Forward	5'-GTGGCCCGGATGTGAGAAG-3'
	Reverse	5'-GGAGCCCTTGTCGGATGATG-3'
GAPDH	Forward	5'- GGCATCTTGGGCTACACT-3'
	Reverse	5'- GCCGAGTTGGGATAGGG-3'
RNASE4	Forward	5'- GCTGTCGACCAGTGTCAAG -3'
	Reverse	5'- GCAGCCCAGGCAAAGAA -3'
Lnc-EGFR-1 (RIP)	Forward	5'- CTCCGGAGGGAGATAATGGT -3'
	Reverse	5'- ATGGCATGTACCAGCGATTC -3'
Lnc-EGFR-2 (RIP)	Forward	5'- GCATTTCTGGGAGGCTCT -3'
	Reverse	5'- GGCCAGGATGGCATGTA -3'
Control (RIP)	Forward	5'- GCAGAACAGCTGACACCA -3'
	Reverse	5'- TGGAAGGAAACCTCAAATCCA -3'
Lnc-EGFR siRNA-1	5’- GCTCTGCTTTAGTCAGGGT -3’	
Lnc-EGFR siRNA-2	5'- TACATGCCATCCTGGCCAT -3'	
EGFR siRNA-1	5’-GGCTGGTTATGTCCTCATT -3’	
EGFR siRNA-2	5’-CAAAGTGTGTAACGGAATA -3’	

Supplementary Table 2 Probe numbers and ENST names

ProbeName	lncRNA ID	Chrosome	Strand	Start	End	cdsstart	cdsend
p9947	ENST00000417926.1	2	+	110693918	110726154	110726154	110726154
p11916	ENST00000481773.1	3	+	113933159	113954241	113954241	113954241
p3251	ENST00000536412.1	12	-	66645524	66651214	66651214	66651214
p19378	TCONS_00022866	14	-	98426422	98444457	98444457	98444457
p1739	ENST00000420367.1	10	-	112629625	112631991	112631991	112631991
p3328	ENST00000550287.1	12	-	94101566	94131599	94131599	94131599
p4971	ENST00000499910.2	14	+	97925206	97930496	97930496	97930496
p4370	ENST00000554286.1	14	-	21161638	21175279	21175279	21175279
p5215	ENST00000602360.1	15	-	66780995	66781427	66781427	66781427
p14995	ENST00000437763.2	7	-	80000501	80003755	80003755	80003755
p2549	ENST00000532350.1	11	-	122026129	122073770	122073770	122073770
p9977	ENST00000590516.1	2	+	118754299	118770258	118770258	118770258
p3217	ENST00000554476.1	12	-	57540272	57541312	57541312	57541312
p7047	ENST00000580169.1	17	-	73161680	73163344	73163344	73163344
p15953	ENST00000521490.1	8	-	132142634	132157128	132157128	132157128
p7525	ENST00000585369.1	17	+	75465149	75467127	75467127	75467127
p10746	ENST00000417335.1	21	-	40360632	40378079	40378079	40378079
p3263	ENST00000546086.1	12	-	68727032	68835996	68835996	68835996
p7231	ENST00000578585.1	17	+	20691383	20692897	20692897	20692897
p3244	ENST00000535315.1	12	-	65893470	66035937	66035937	66035937
p3296	ENST00000550926.1	12	-	74931924	74932413	74932413	74932413
p7475	ENST00000592030.1	17	+	66409880	66424904	66424904	66424904
p1229	ENST00000456633.1	1	+	155531832	155533437	155533437	155533437
p5486	ENST00000559867.1	15	+	34547486	34550033	34550033	34550033
p29432	ENST00000434593.1	6	-	134846462	134980974	134980974	134980974
p3451	ENST00000512624.2	12	-	127399765	127544942	127544942	127544942
p28262	ASO1952	None	None	None	None	None	None
p29328	ENST00000449424.1	22	+	21821679	21825558	21825558	21825558

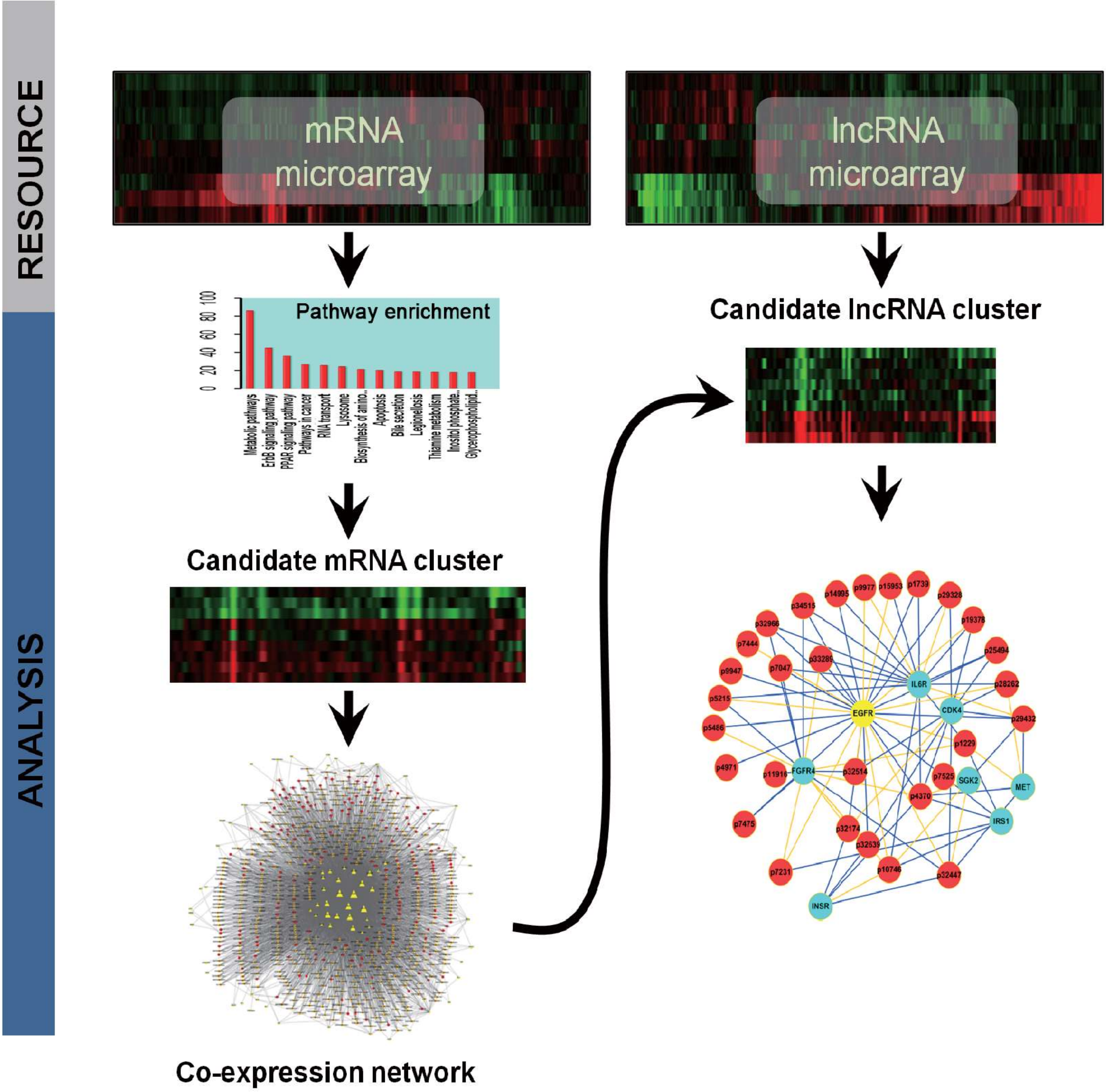

Supplementary Figure 1. A schematic overview of the screening workflow based on the high throughput detection.

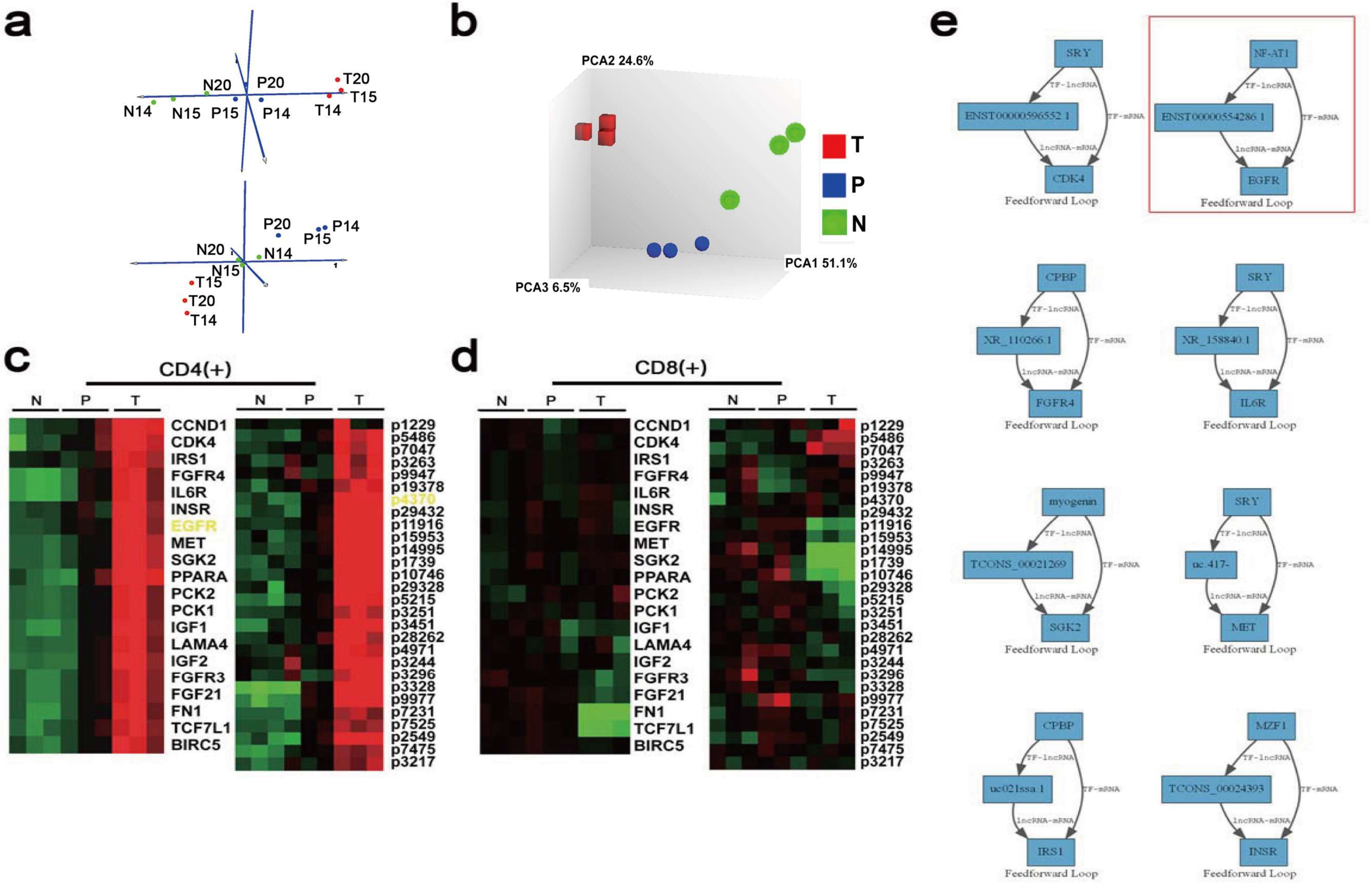

Supplementary Figure 2. The candidate feed-forward loops after phase I validation based on microarray detection.

(a) PCA analysis of the nine samples by using the expression profiling of mRNA or lncRNA separately, the upper panel indicated mRNA while the lower is lncRNA. **(b)** The merged PCA analysis of nine samples in microarray detection. **(c, d)** Relative expression level of candidate mRNA and lncRNA after phase I validation in CD4 (+) and CD8 (+) T cell respectively. Data was presented as heatmap with log-transformed. N: PBL-C; P: PBL-T; T: TIL-T. **(e)** The predicted feed-forward loops.

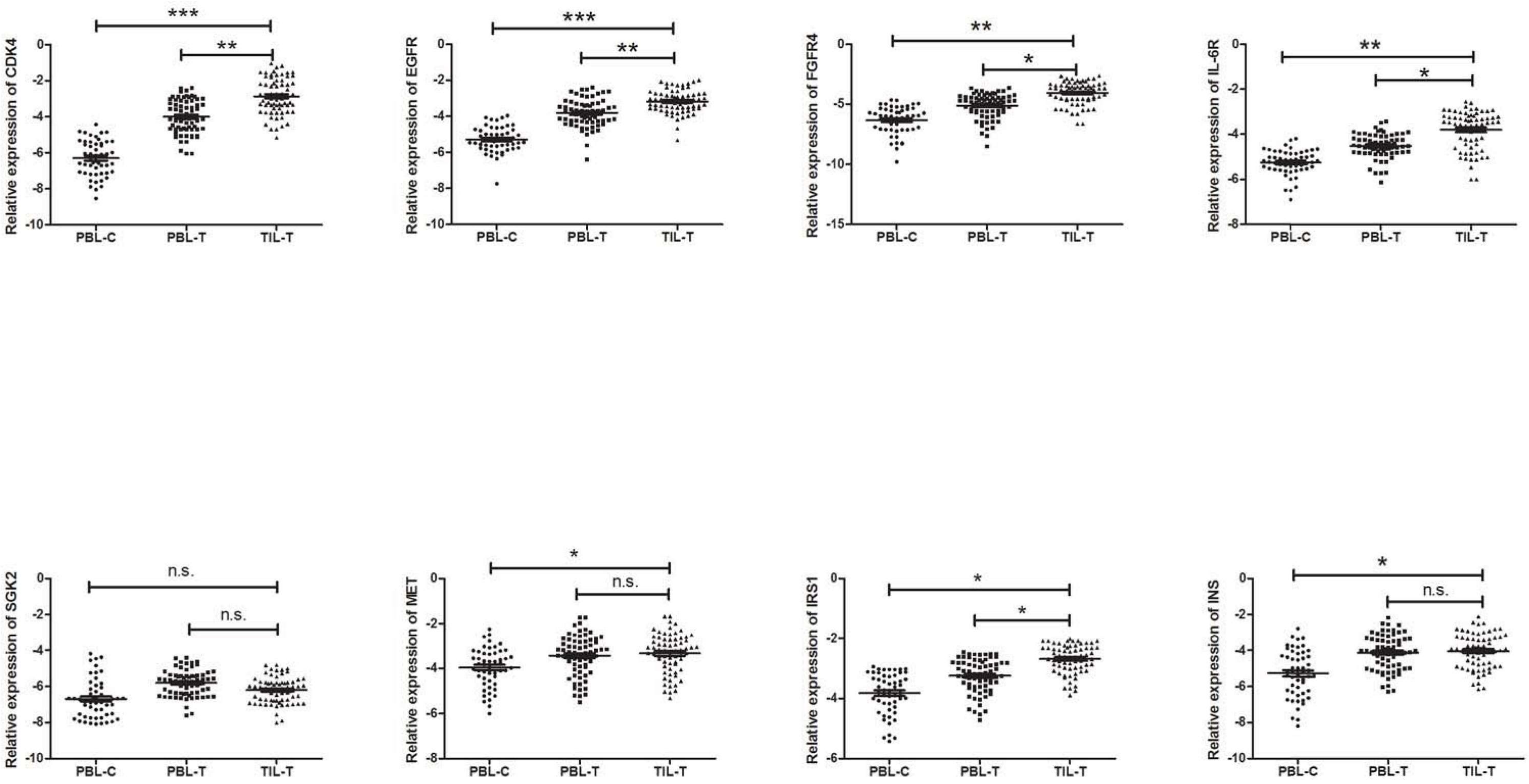

Supplementary Figure 3. Phase II validation of candidate mRNA in 67 pairs of TIL and PBL obtained from HCC patients and 52 PBL of healthy controls.

Data was log-transformed as presented with mean ± SEM (n.s. indicated no significance; * indicated *P*<0.05; ** indicated *P*< 0.01; *** indicated *P*<0.001).

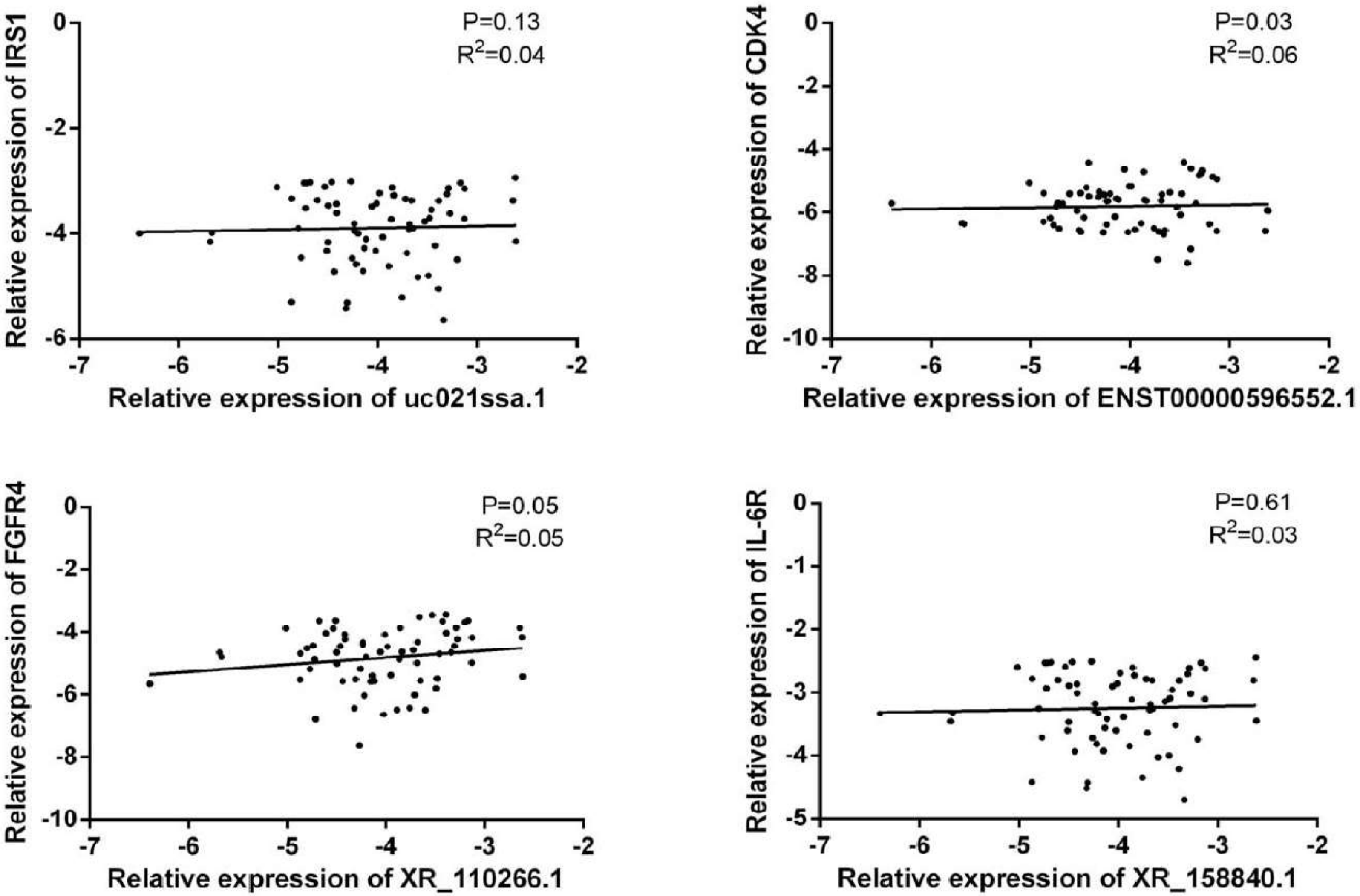

Supplementary Figure 4. Pearson analysis of lncRNA and mRNA in predicted feed-forward loops. Data is log-transformed as presented with mean ± SEM. P value and R^2 value is presented in the top right corner.

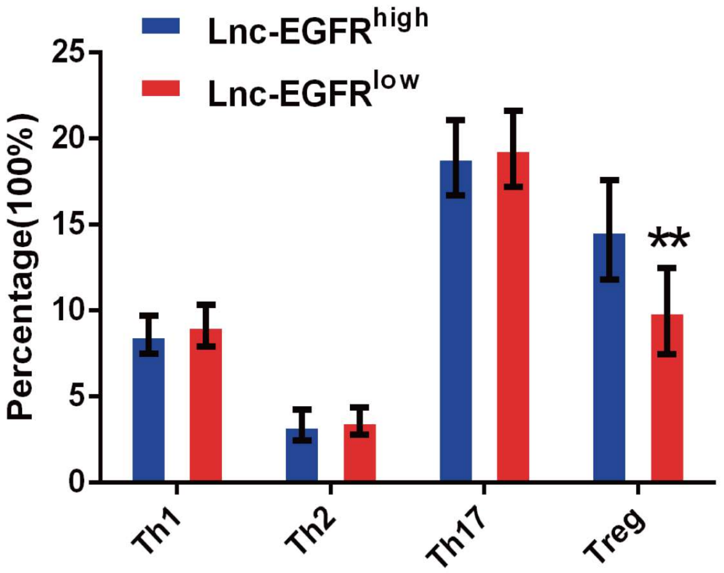

Supplementary Figure 5. The distribution of CD4 (+) T cell subgroup based on different expression of lnc-EGFR

The expression level of lnc-EGFR was divided by using the upper 95% CI of lnc-EGFR in PBL-C as cutoff. The percentage of Th1, Th2, Th17 and Treg was examined by flow cytometry. Data are presented as mean ± SEM.** indicated *P*<0.01.

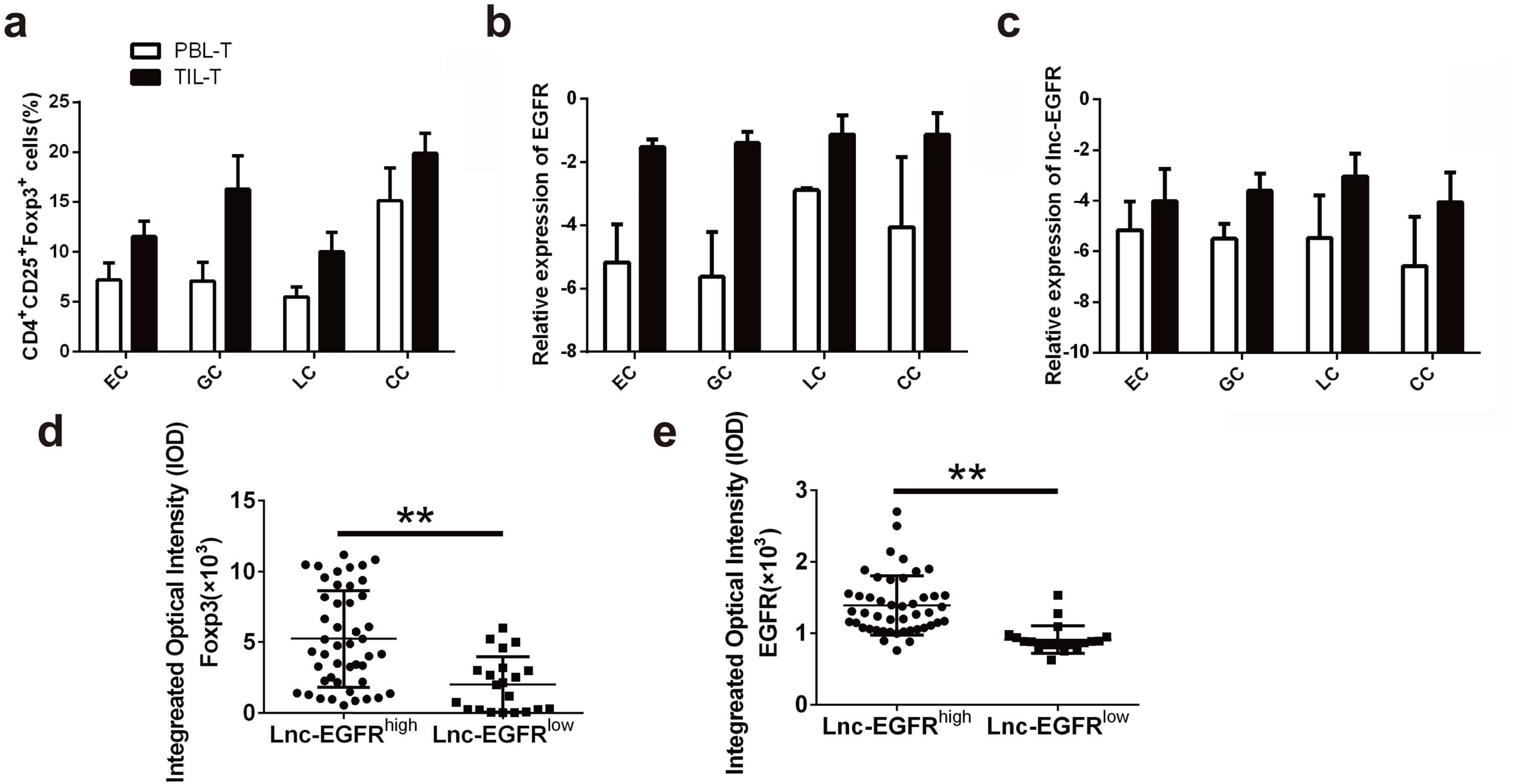

Supplementary Figure 6. Lnc-EGFR and EGFR expression in human samples. **(a)** The distribution of Treg in TIL-T of patients with other malignant tumor comparing with the PBL-T samples by flow cytometry. **(b)** Relative expression of EGFR inTIL-T of patients with other malignant tumor comparing with the PBL-T samples. **(c)** Relative expression of lnc-EGFR inTIL-T of patients with other malignant tumor comparing with the PBL-T samples. **(d)** Integral optical density (IOD) analysis of EGFR stain. **(e)** Integral optical density (IOD) analysis of Foxp3 stain. Data are presented as means ± SEM and analyzed with Student t test (** P < 0.01). EC: Esophageal cancer; GC: Gastric cancer; LC: Lung cancer; CC: colon cancer.

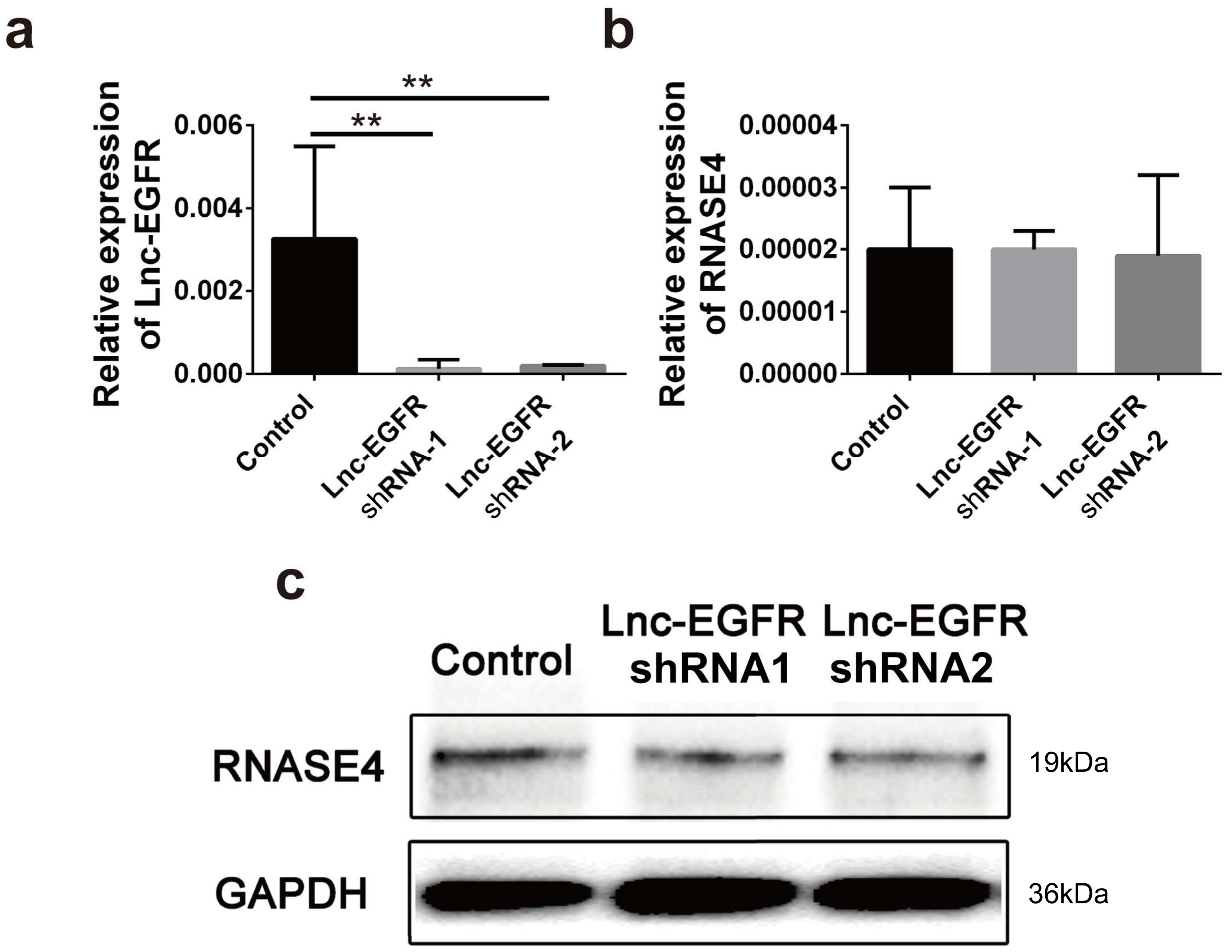

Supplementary Figure 7. Lnc-EGFR shRNAs have no effect on expression of RNASE4

(a) Relative expression of lnc-EGFR after treating with lnc-EGFR shRNA. **(b)** Relative expression of RNASE4 after treating with lnc-EGFRshRNA. **(c)** Protein expression of RNASE4. Data are presented as means ± SEM and analyzed with Student t test (* $P < 0.05$, ** $P < 0.01$).

a

Accession	Description	Score	# AAs	MW [kDa]
29725609	epidermal growth factor receptor isoform a precursor [Homo sapiens]	25.08	1210	134.2

Sequence	XCorr	Charge	m/z [Da]	MH+ [Da]	First Scan
ELVEPLTPSGEAPNQALLR	2.23	2	1017.54529	2034.08330	12905

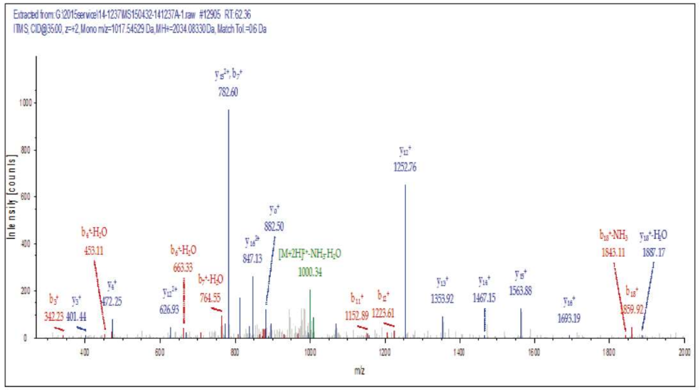

b

#	Protein region	RNA region	Interaction Propensity	Discriminative Power	Normalized Score
1	1001-1052	340-391	17.12	47	3.68
2	1001-1052	326-377	15.03	42	3.25
3	997-1048	340-391	14.85	40	3.21
4	122-173	340-391	14.51	40	3.14
5	997-1048	576-627	12.61	35	2.74
6	122-173	576-627	12.59	35	2.74
7	1001-1052	401-452	12.45	35	2.71
8	1001-1052	426-477	12.25	35	2.67
9	126-177	340-391	12.12	35	2.64
10	1001-1052	315-366	11.72	33	2.56

c

CGGCAGAACAGCTGACACCACCAACTTTAAAGAGCCCAACTGATGAACTGATCAG
CATGAGAATACTGTTTTTTCATCTCTCTGTCCCATGACTTCACTCTGCATTCTTCAA
CCAATTAGTGATCTCTTATTTCCACCTACTCCAAATCCTTAAAAACAACAGCCTCAA
ACTCTTTGAGGAAATGGATTTGAGGTTTCCTTCCATCTTTTGGTTCAGCAGCCCTG
CAATTAAACCTCTTTCTCTGCTGCAACCCAGTGTCTTGGCATATTGACATGCTGTG
CACATCAGGCAAAAGACCTATTACCATTACATGAGGACCCCTCCGGAGGGAGATA
ATGGTGGGGGCATTTCTGGGAGGCTCTGCTTTAGTCAGGGTGCACGTGTTGCCGC
AGGAATCGCTGGTACATGCCATCCTGGCCATAGGAGGGCTGGACCAGCCCCAGC
CCCAGCAGGGTCAGCAGCAAAAGCAGAAGCAATGAATGGGTCCTCTGCAGAGCC
ATCAGTATCTTAGAGGTGCCTAGAAAAGAAAGCAAGGGGCAGGAGAAATAAGAG
AGAAGTTGCTGTGGCCAGTACAATGACACAGGGCAGGGACTGACTGCGTGGCTG
CCAGGTTTGTGTGGACAGAGTATAAAAAGAGAGAAAAGGGTGAGGTCAGGCCCCGTC
TCCTCATGATGCTTTCACAGTATCTGTCATCCCGGCCCTGTGGTTTGGCATCATAGTGC
TGGGTCAGGAAGTGTGTGTACCTGGAGTTATCCTGAGCCAGGGTCGGTGGGGTC
AGACCCAGACCCAGCACGAAGACCAACAACAAAACGCCCAGGCCCATCACCATCTCTTCC
AACACAGGCTCCTGCGGAGGGCAAAAGGAGGTGTGGTAGACCCAAGAACAGCATCACCAAA
TTAGGCCAAAAGGACAG

R1-1 R1-2 R2 R3

d

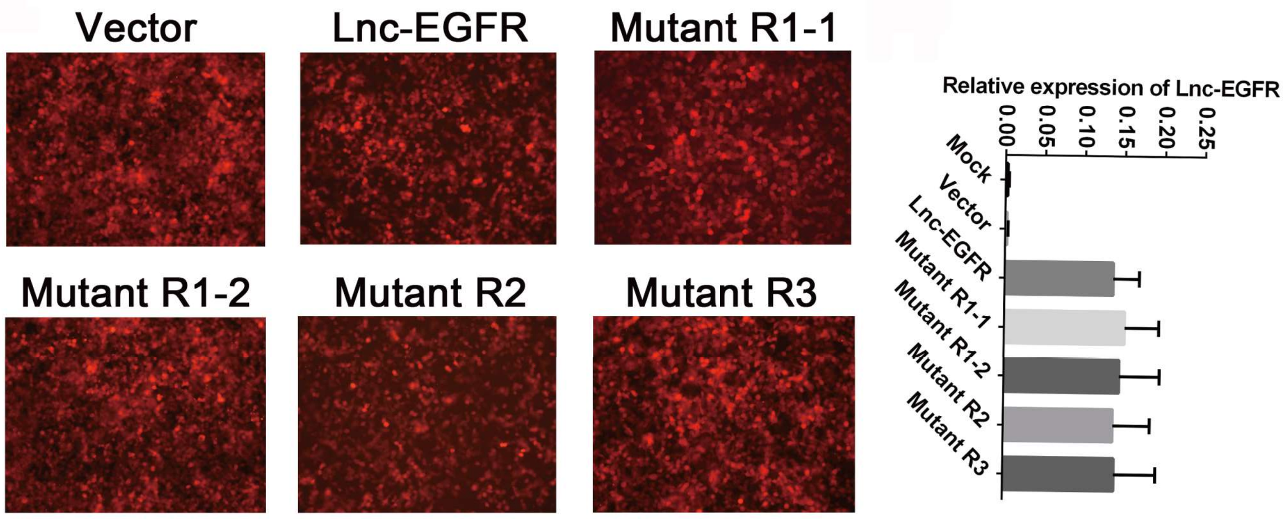

Supplementary Figure 8. Binding site design and overexpression validation.

(a) The sequence of peptide detected by mass spectrometry, the lysine residue presented as secondary ion mass spectrometry.

(b) Binding site prediction between lnc-EGFR and EGFR.

(c) Detailed sequence design of mutation type.

(d) Fluorescence intensity of cells overexpressed with different condition.

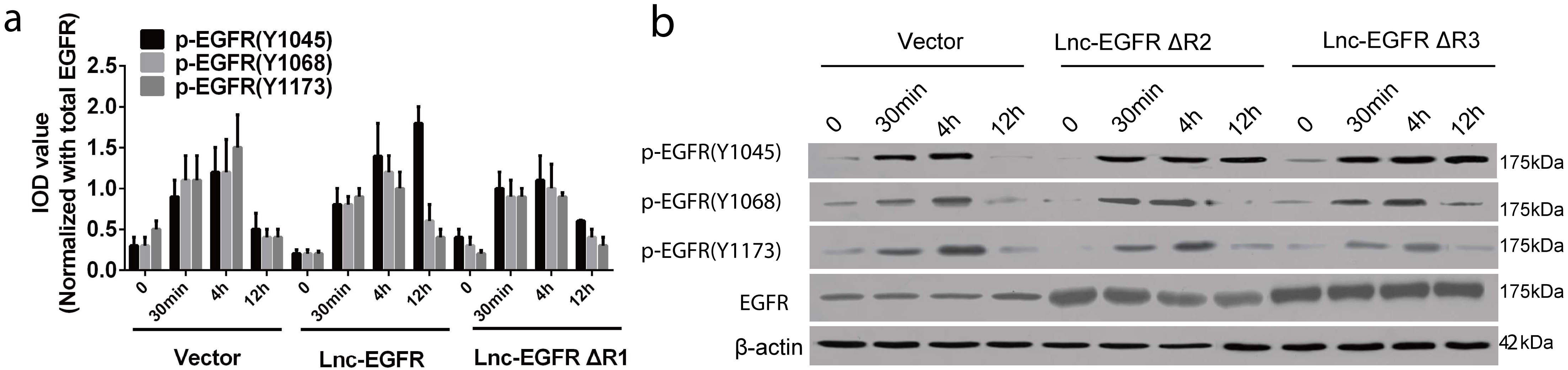

Supplementary Figure 9. Increased lnc-EGFR sustains EGFR phosphorylation through binding with EGFR in Region 1 with a suppression of PI3K/AKT . (a) The IOD analysis of EGFR phosphorylation normalized with total EGFR expression. (b) The expression of phosphorylated EGFR and total EGFR in cells treated with lnc-EGFRΔR2 and lnc-EGFRΔR3.

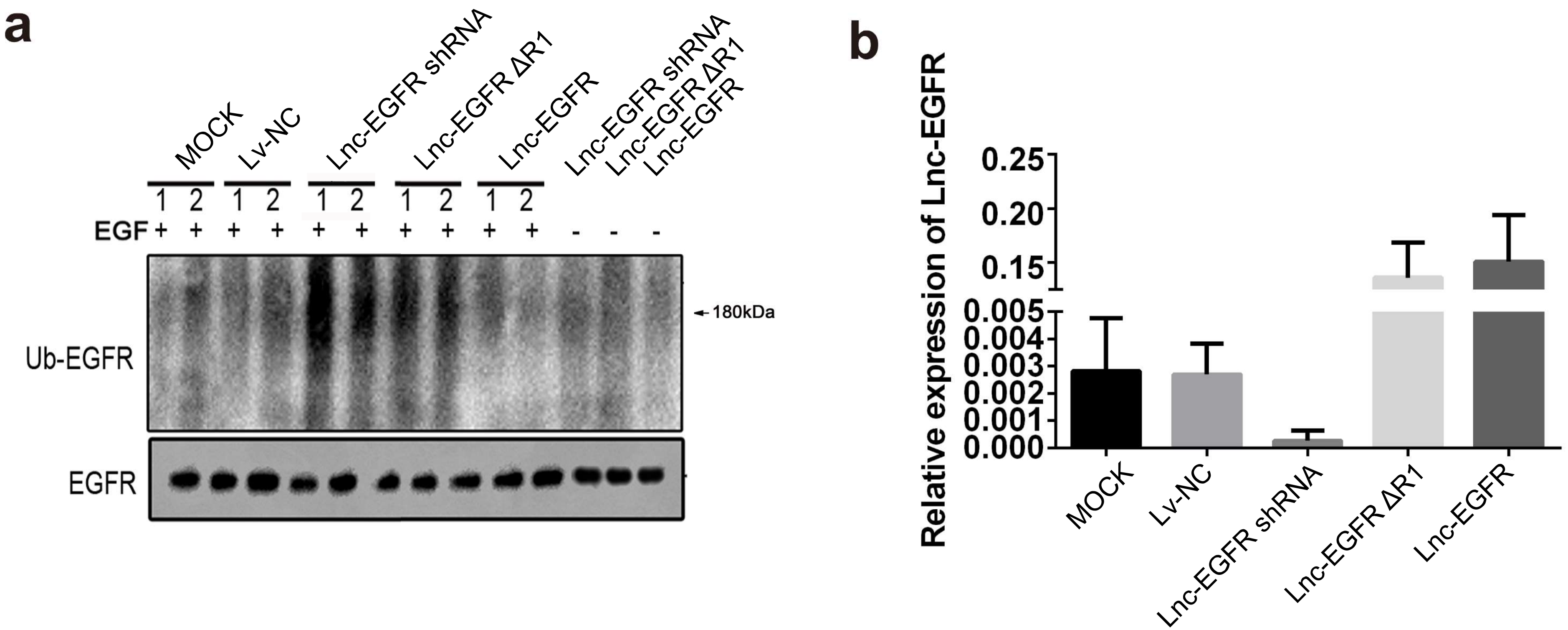

Supplementary Figure 10. LncEGFR inhibits EGFR ubiquitination in response to EGF.
(a) Ubiquitination of EGFR in human HCC patient T cells transduced with indicated lentiviral particles in the presence or absence of EGF (100 ng/ml for 90 minutes) was detected by IP/IB using anti-EGFR antibodies and anti-ubiquitin antibodies, respectively (upper panel). The equal loading of EGFR protein was determined by IB using anti-EGFR antibodies (lower panel).
(b) Relative expression of LncEGFR in each condition in (a) was assessed by qPCR.

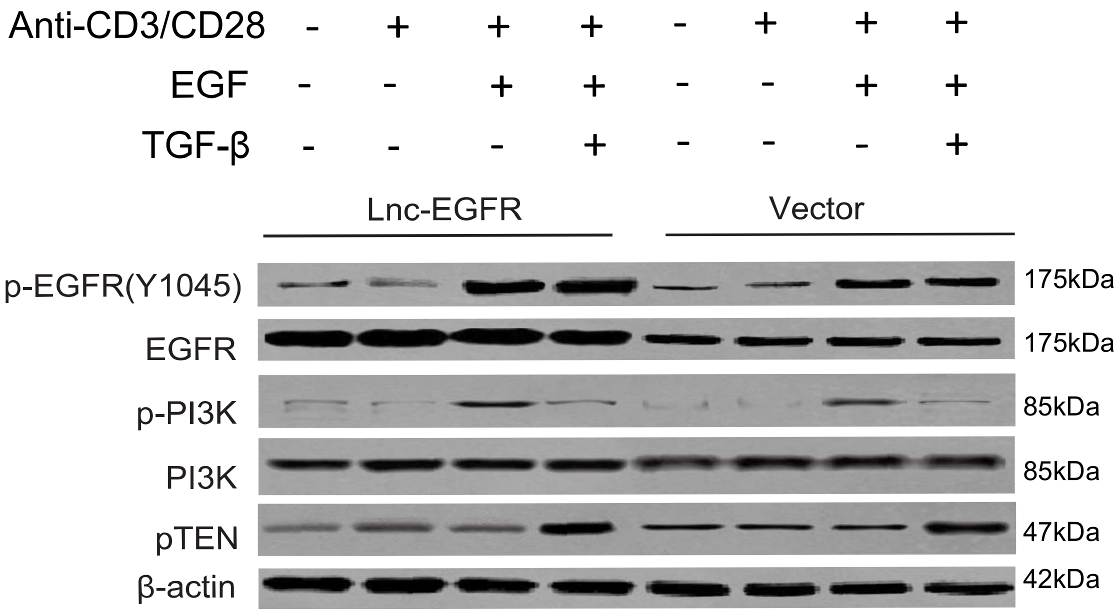

Supplementary Figure 11. Increased lnc-EGFR have no effect on PI3K/AKT.
Conventional T cell was activated in vitro by anti-CD3, CD28 beads, the activation of EGFR/PI3K signaling was detected in both vector and lnc-EGFR group treated with , the expression of EGFR increased in conventional T cell with overexpression of TGF-β.

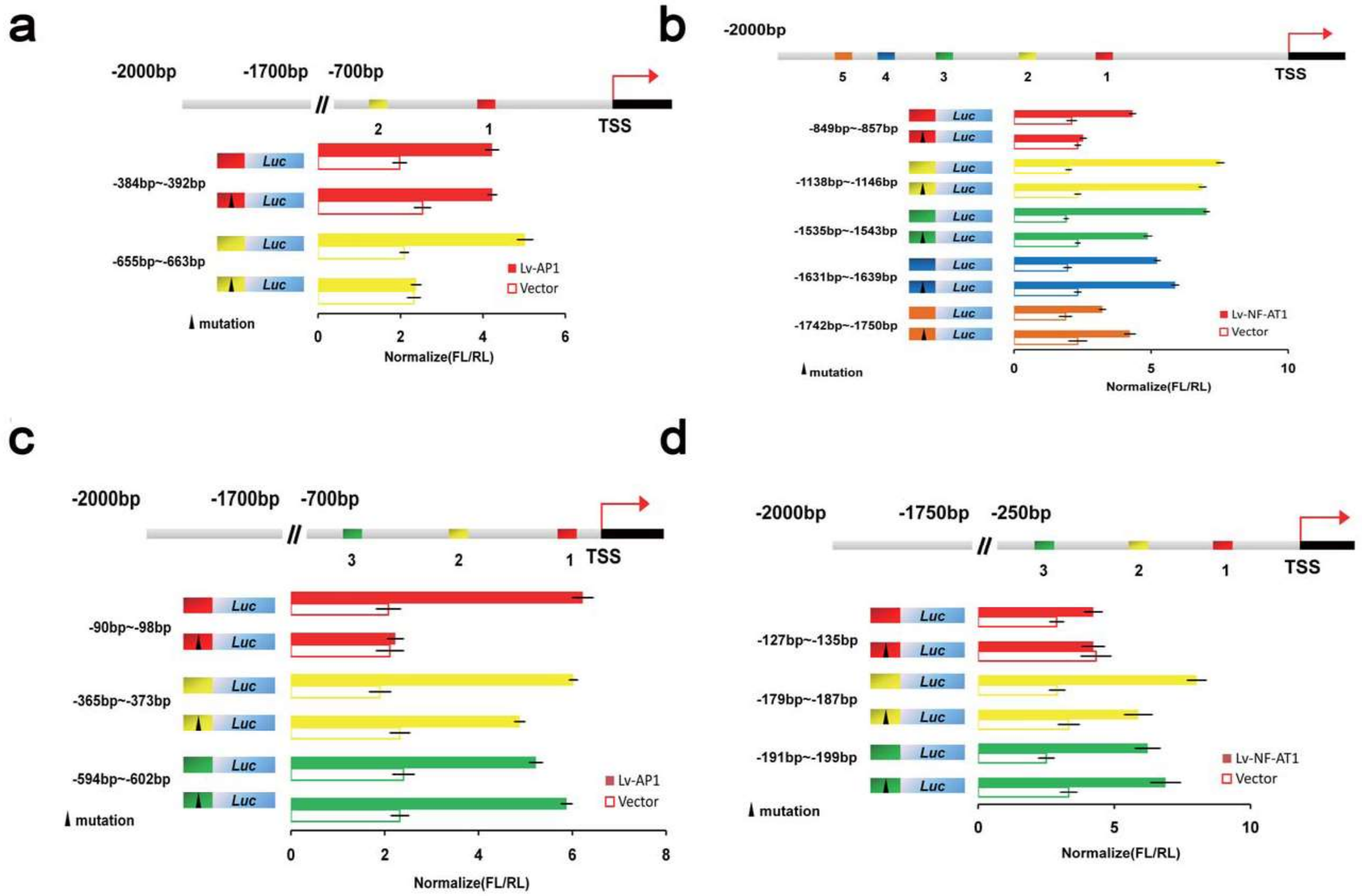

Supplementary Figure 12. Potential binding site screening of NF-AT1 and AP1 in the promoter region of EGFR and lnc-EGFR respectively.

The schematic axis indicates the potential binding site of transcription factors on target genes and is presented in the top o f each panel. pGL4 vector cloned with either wild type or the mutant binding site was electrotransfected in CD4 (+) cells accompanying with Ranilla. The binding site was shown in the lower left while the Δ indicated the mutant type. Data was presented with Firefly luciferase(FL) by normalizing with Ranilla luciferase(RL). **(a)** AP1/EGFR; **(b)** NF-AT1/EGFR; **(c)** AP1/lnc-EGFR; **(d)** .NF-AT1/lnc-EGFR

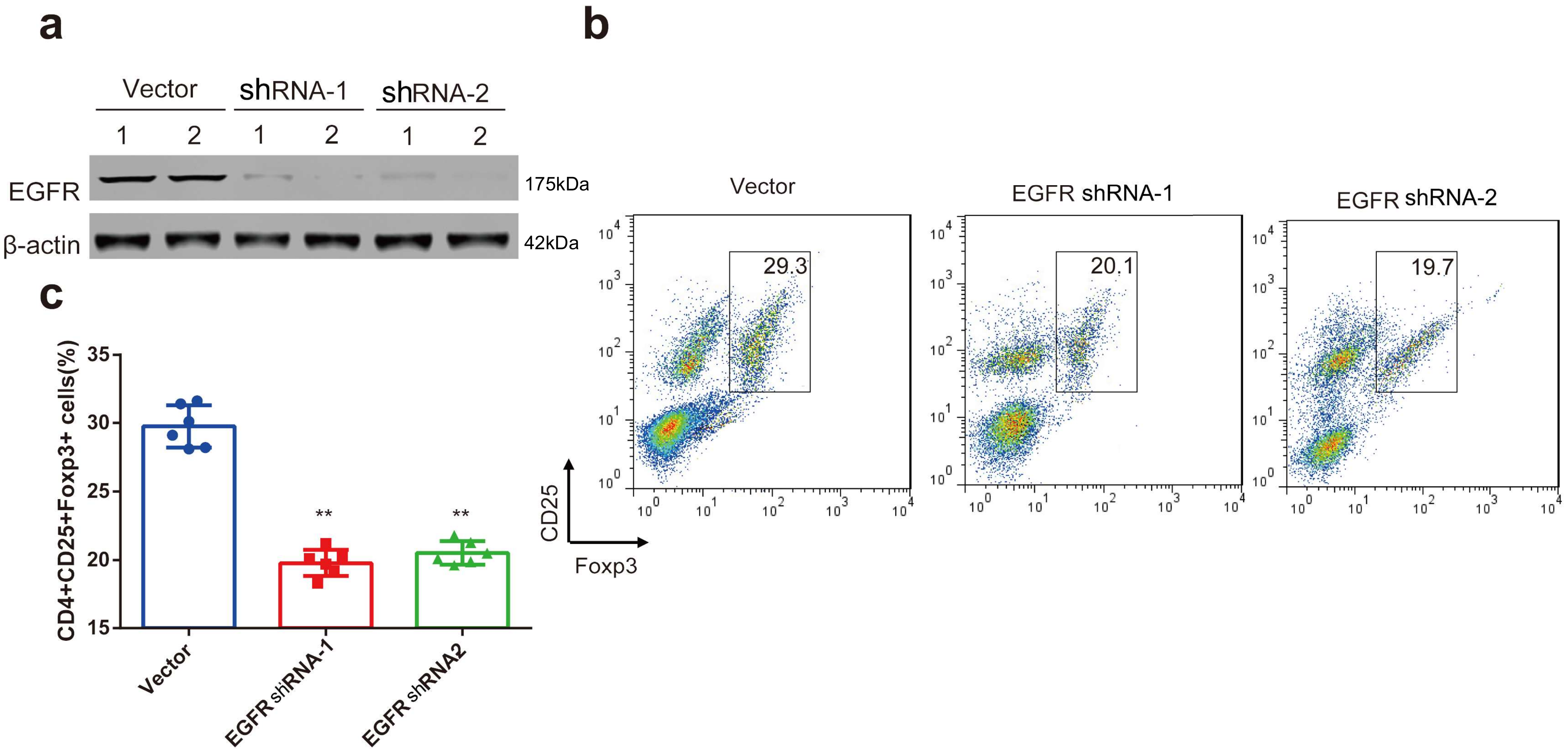

Supplementary Figure 13. Decreased EGFR impairs the percentage of Treg cells. **(a)** Protein expression of EGFR in T cells treated with EGFR shRNAs. **(b)** The distribution of Treg labelled by CD4/CD25/Foxp3 through flow cytometry. **(c)** The percentage of Treg cells in different groups. Data are presented as means ± SEM and analyzed with Student t test (** $P < 0.01$) .

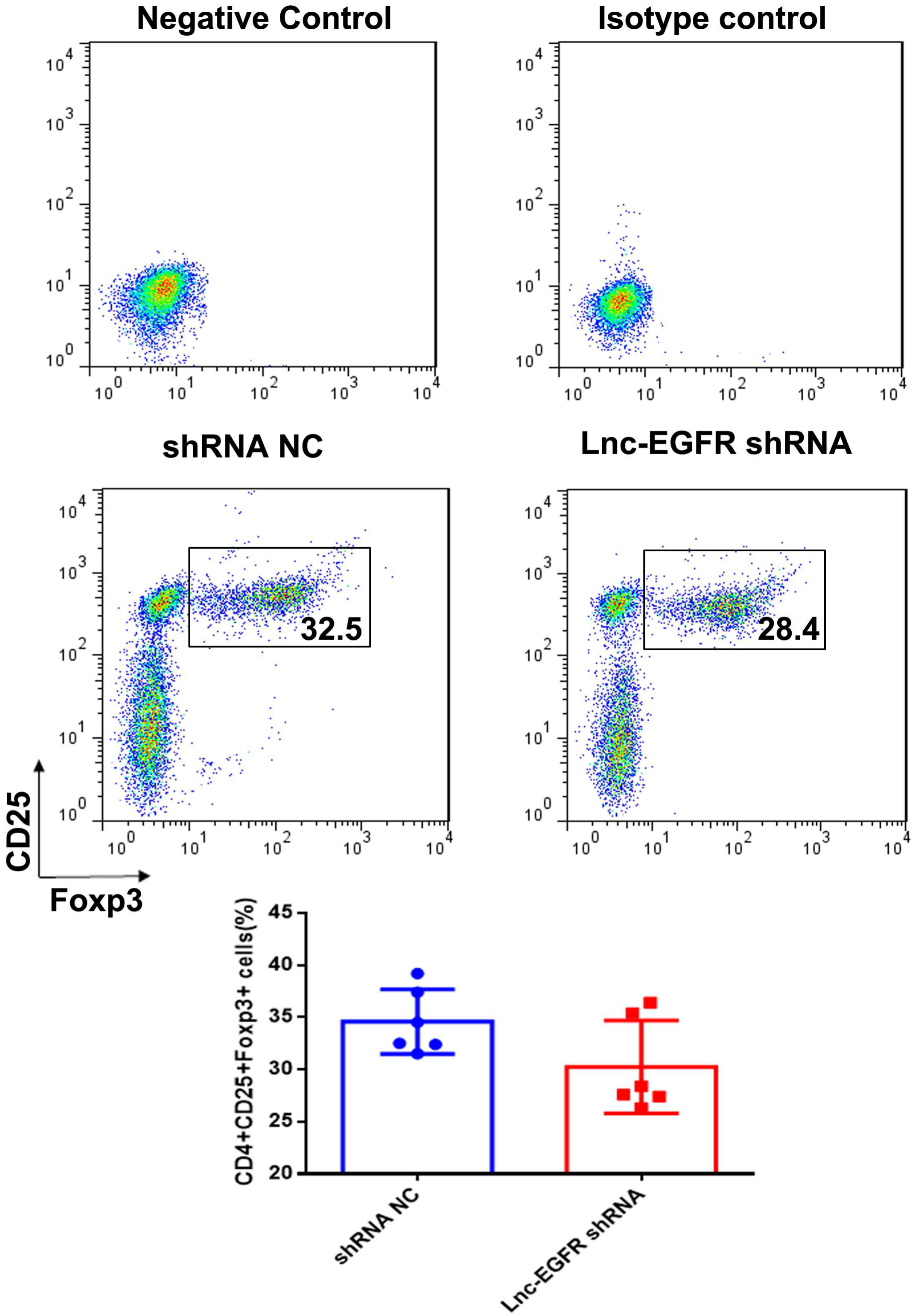

Supplementary Figure 14

T cells isolated from the PBMCs of healthy controls transduced variously indicated in the figure and polarization stimulation to Treg cells.

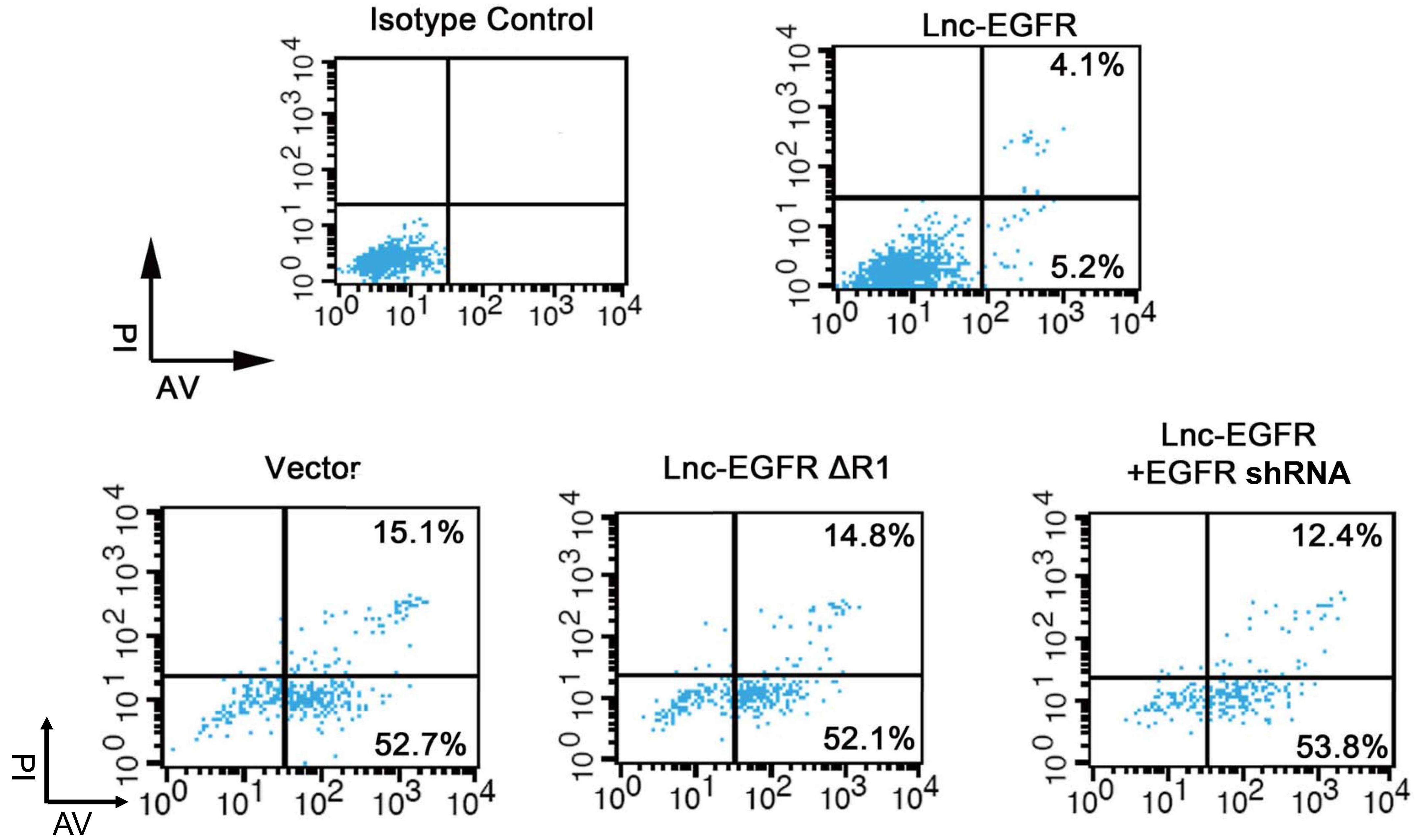

Supplementary Figure 15 . Lnc-EGFR inhibits the apoptosis in tumor cells
The apoptosis of tumor cells was analyzed by FCM with PI/Annexin V-FITC. Lnc-EGFRΔR1 indicated cells treated with lentivirus overexpression mutanted binding site of R1 in lnc-EGFR.

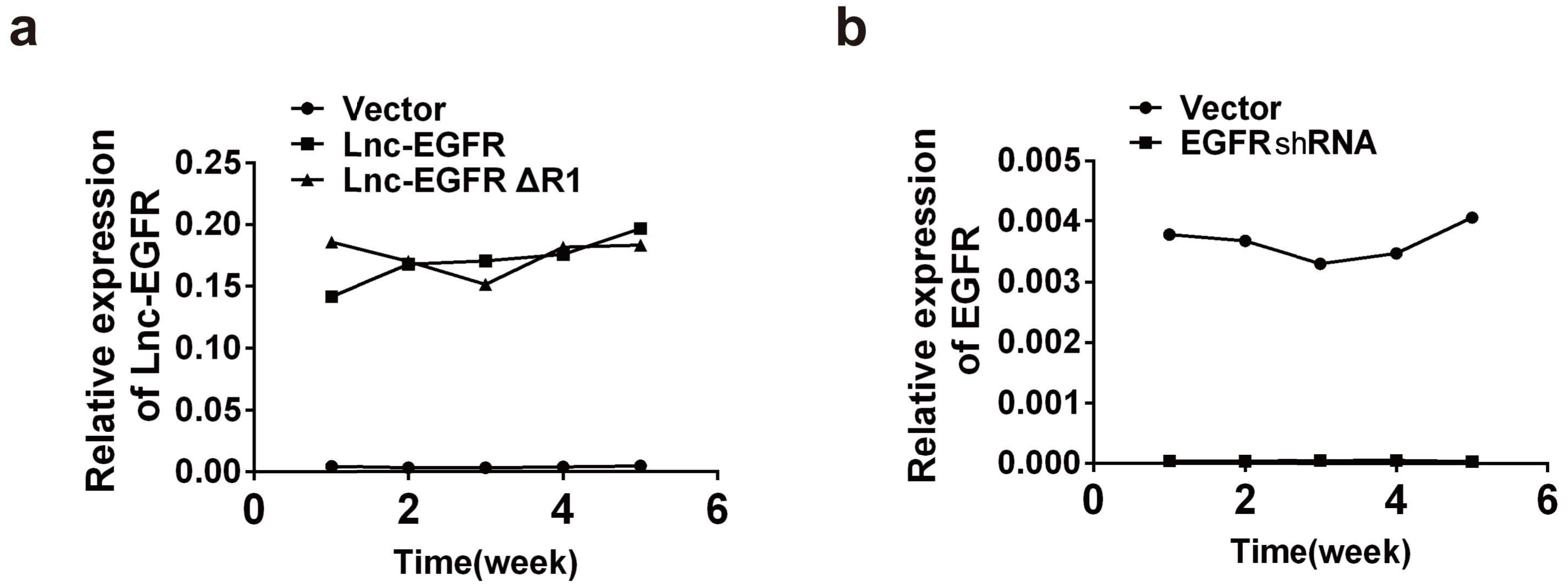

Supplementary Figure 16. Time based expression of lnc-EGFR and EGFR over the time-course of the tumor study. **(a)** Relative expression of lnc-EGFR. **(b)** Relative expression of EGFR.

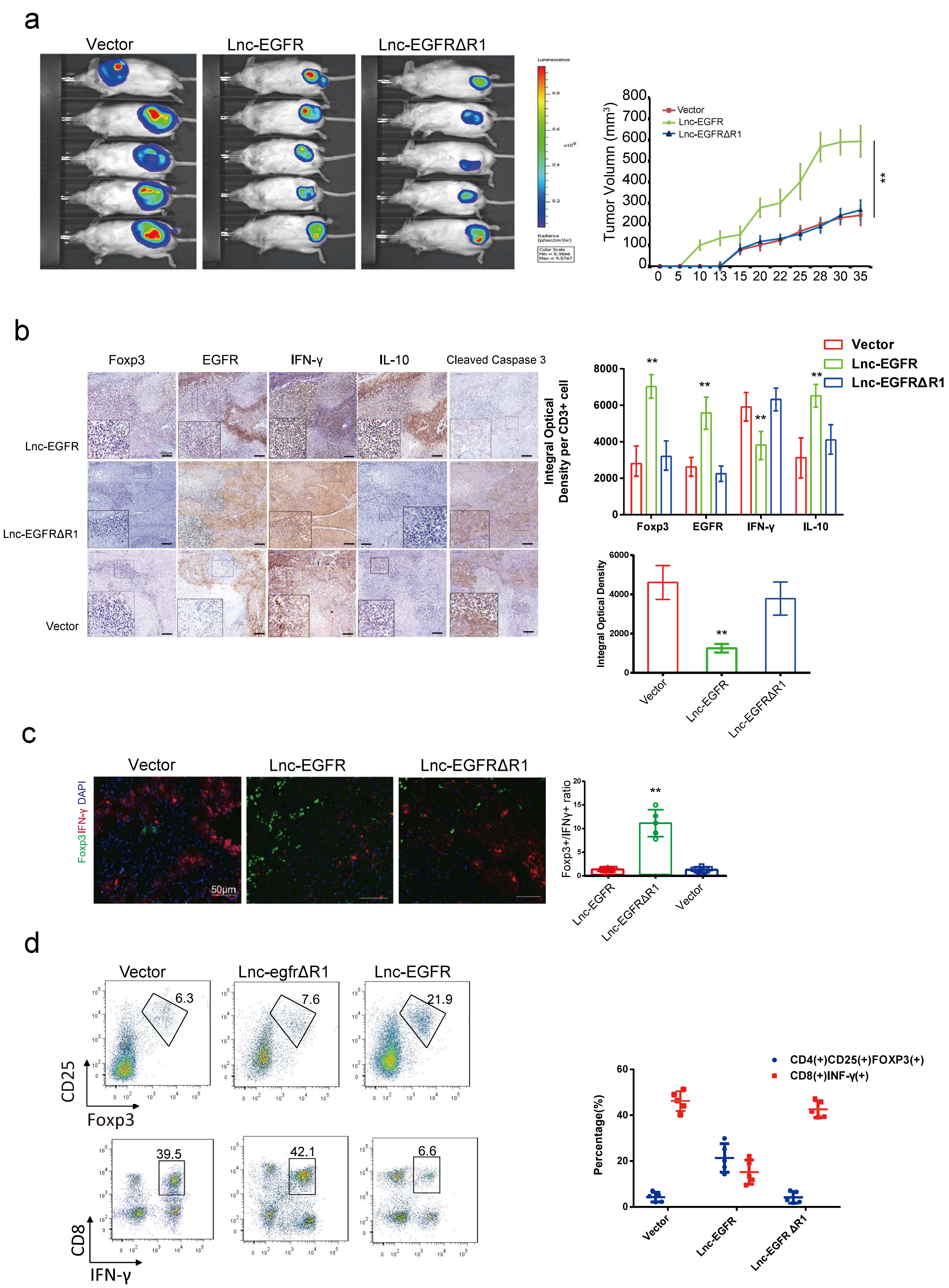

a
Vector
Lnc-EGFR
Lnc-EGFRΔR1
Vector
Lnc-EGFR
Lnc-EGFRΔR1
Tumor Volumn (mm³)
**
b
Foxp3
EGFR
IFN-γ
IL-10
Cleaved Caspase 3
Lnc-EGFR
Lnc-EGFRΔR1
Vector
Integral Optical Density per CD3+ cell
Vector
Lnc-EGFR
Lnc-EGFRΔR1
Integral Optical Density
c
Vector
Lnc-EGFR
Lnc-EGFRΔR1
Foxp3 IFN-γ DAPI
50μm
Foxp3+/IFNγ+ ratio
d
Vector
Lnc-egfrΔR1
Lnc-EGFR
6.3
7.6
21.9
CD25
Foxp3
39.5
42.1
6.6
CD8
IFN-γ
CD4(+)CD25(+)FOXP3(+)
CD8(+)INF-γ(+)
Percentage(%)
Vector
Lnc-EGFR
Lnc-EGFR ΔR1

Supplementary Figure 17. Lnc-EGFR enhances tumor growth in vivo

(a): Mice with established tumors in different groups were imaged every 7 days with the IVIS Lumina II system; the images shown were taken on day 25. Tumor growth was measured approximately every 3 days and is presented in the right panel. (b): Immunohistochemistry was performed in paraffin sections from the xenograft tumors (n=6 for each group) (×100). The integral optical density (IOD) of the T cells was calculated by comparing them with $^{+}$CD3 cells in paired sections, and the IOD of cleaved caspase 3 was calculated in the tumor cells and is presented in the right panel(c): Foxp3 and IFN-γ were detected in biopsy samples obtained from the tumor tissues of the in vivo model; the green stain indicates Foxp3 and the red stain indicates IFN-γ. DAPI was used to stain the nuclei(×200). Quantitative results are listed on the right, as means ± SEM (n = 5 in each group). (d): The distributions of Tregs and CTLs were determined with flow cytometry in T cells extracted from the tumors of the in vivo model. Data are presented as means ± SEM (*P < 0.05, **P < 0.01).

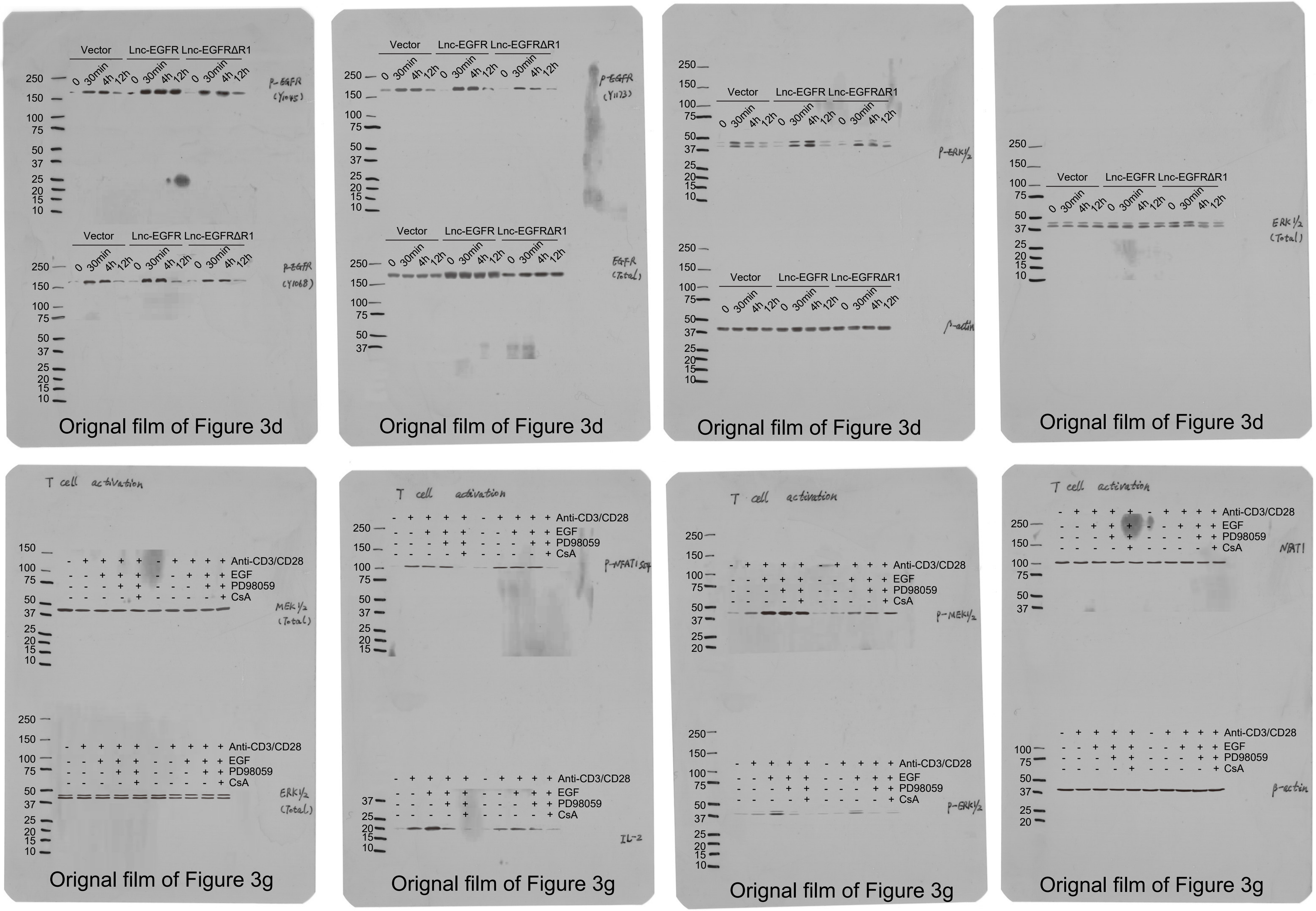

Supplementary Figure 18. Uncropped images of blots in Figure 3-4 and Supplementary Figure 7,9,13 and 11.

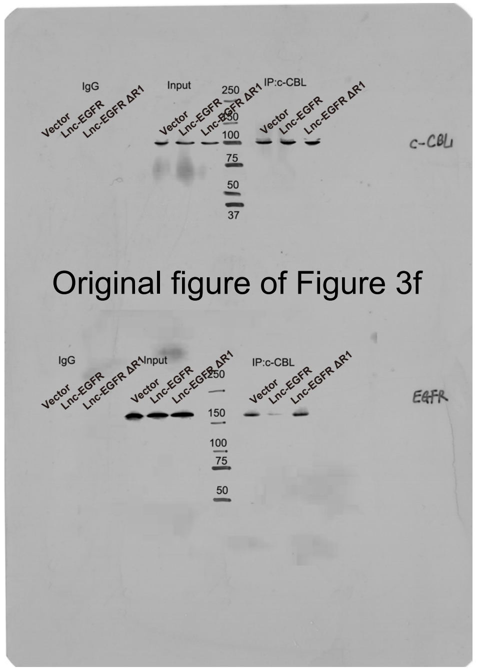

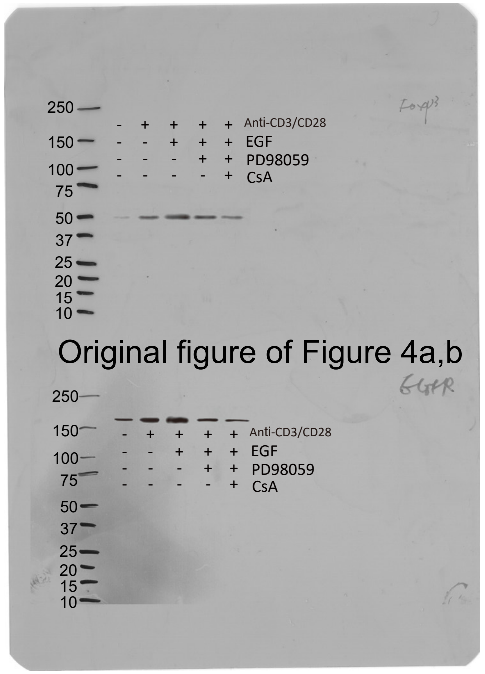

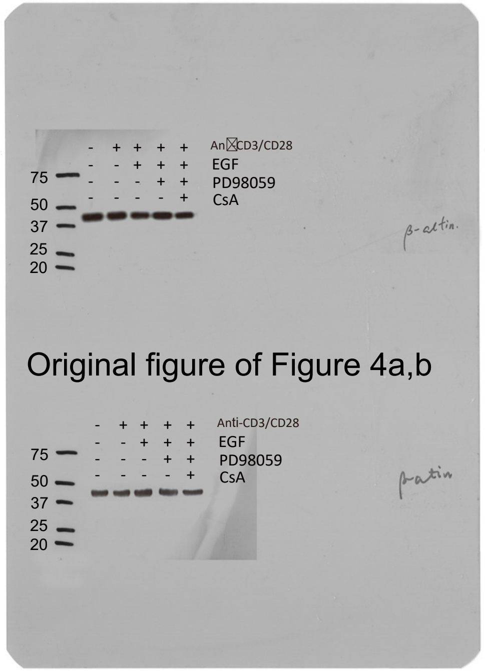

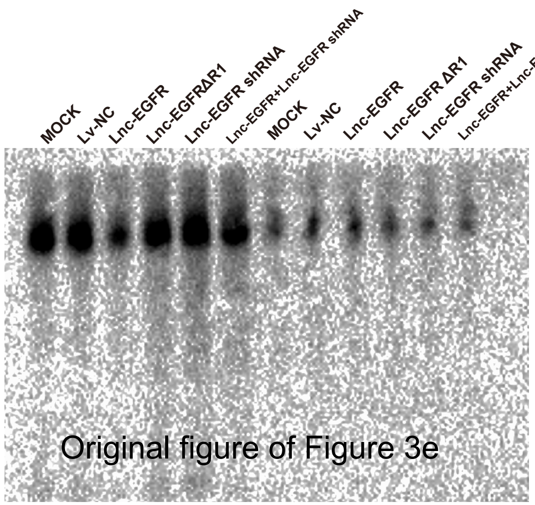

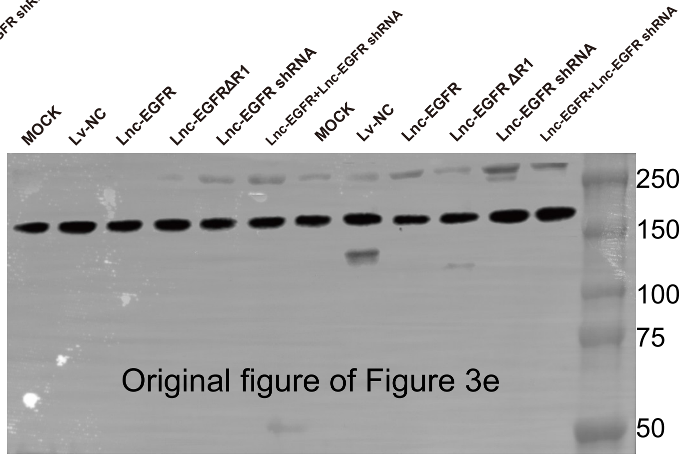

Supplementary Figure 18. Uncropped images of blots in Figure 3-4 and Supplementary Figure 7,9,13 and 11.

Fig. s7c

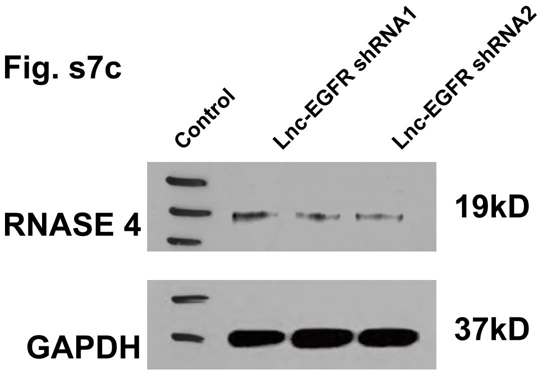

Fig. s11

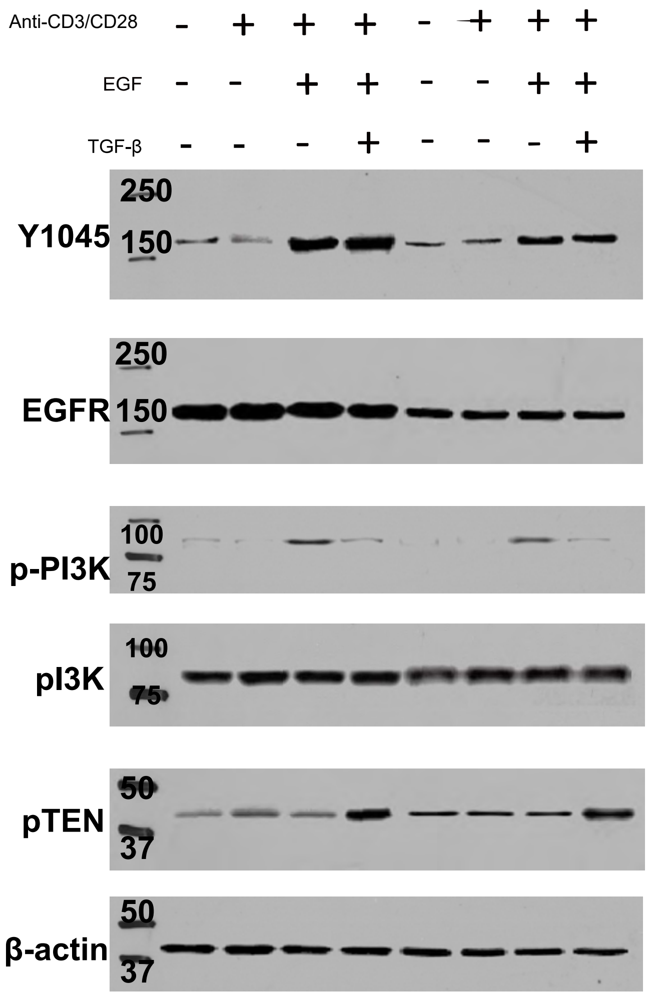

Fig s9b

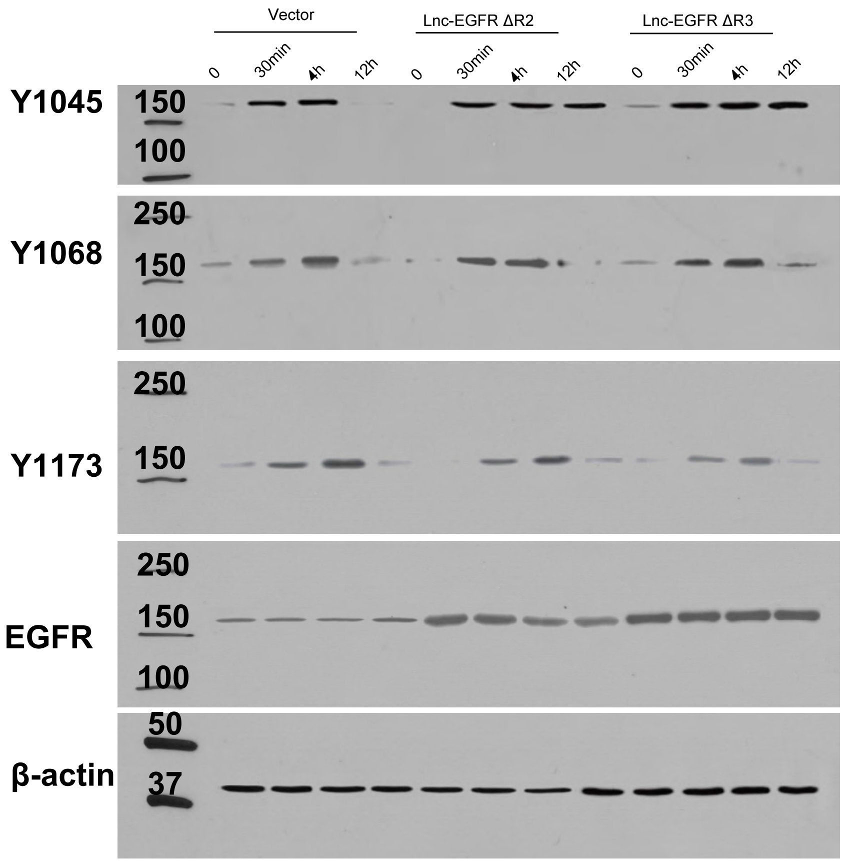

Fig. s13a

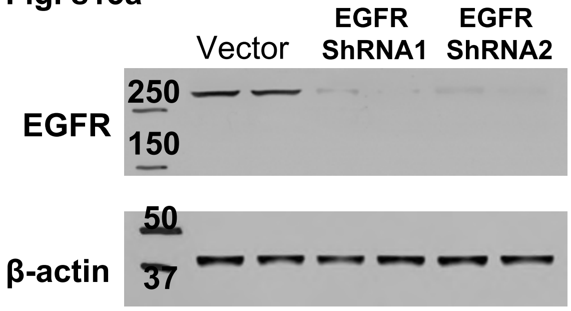

Supplementary Figure 18. Uncropped images of blots in Figure 3-4 and Supplementary Figure 7,9,13 and 11.
